# Quantification of vitamin K (phylloquinone and menaquinones 4–10) in various shellfish

**DOI:** 10.1017/S0007114525000261

**Published:** 2025-02-28

**Authors:** Amalie Moxness Reksten, Kari Elin Rød, Inger Aakre, Lise Madsen, Kristin Holvik, Sigrun Henjum, Eystein Oveland, Lisbeth Dahl

**Affiliations:** 1 Institute of Marine Research, P.O. Box 2029 Nordnes, 5817 Bergen, Norway; 2 The Department of Biomedicine, University of Bergen, Bergen, Norway; 3 Department of Clinical Medicine, University of Bergen, Bergen, Norway; 4 Department of Physical Health and Ageing, Norwegian Institute of Public Health, Oslo, Norway; 5 Department of Nursing and Health Promotion, Faculty of Health Sciences, Oslo Metropolitan University, Oslo, Norway

**Keywords:** Scallop, Shrimp, Blue mussels, Crab, Lobster, Crayfish

## Abstract

Vitamin K exists naturally in foods as phylloquinone (vitamin K_1_, PK) and as a range of menaquinones (vitamin K_2_, MK). There is scarce information on the occurrence and distribution of PK and MK in dietary sources, particularly in seafood. This study aimed to comprehensively analyse the contents of vitamin K_1,_ dihydro-K_1_, and MK-4 to MK-10 in various species, tissue types and processing degrees of shellfish. Additionally, seasonal differences in the vitamin K content of blue mussels (*Mytilus edulis*) were explored. Most shellfish products had low contents of total vitamin K (< 10 µg/100 g). The highest content of total vitamin K was found in the hepatopancreas of snow crab (170 µg/100 g), the brown meat of brown crab (35 µg/100 g), pre-packaged blue mussels (20 µg/100 g), stuffed brown crab shells (15 µg/100 g) and blue mussels in brine (12 µg/100 g). In general, the hepatopancreas of crustaceans contained considerably higher contents of vitamin K than their white meat counterparts. MK contributed most to total vitamin K contents, whereas most shellfish products contained low contents of PK, thus making only a minor contribution to the adequate intake established for adults. No statistically significant differences were observed in PK and MK contents of blue mussels sampled during spring *v*. late summer (*P* < 0·005). Nevertheless, a non-significant trend of increasing vitamin K content was observed towards the autumn months. This study presents novel vitamin K data for shellfish, an unexplored food group, and adds to the scarce vitamin K composition data worldwide.

Vitamin K is an essential micronutrient in humans and represents a group of fat-soluble structurally related compounds that occur naturally as phylloquinone (vitamin K_1_) and menaquinones (vitamin K_2_). Both forms have a common 2-methyl-1,4-naphthoquinone ring structure, known as menadione, which constitutes the functional group. However, they exhibit disparities in their respective side chains, wherein phylloquinone (PK) is connected to a phytyl group, whereas the side chains of menaquinones are composed of a varying number of unsaturated isoprene units (MK-*n*), where MK-4 to MK-13 have been identified^(1,2)^. PK is synthesised by plants and has historically been considered the predominant dietary form of vitamin K. Dietary sources include green, leafy vegetables and certain plant-derived oils, such as rapeseed and olive oils^(3–5)^. Most MK are formed as a result of bacterial fermentation or by the intestinal microbiota; there is however limited understanding of the extent to which gut bacterial production contributes to human vitamin K status^(1,6)^. Thus, the main sources of MK include foods of animal origin and foods altered by bacterial fermentation, such as fermented foods, meat and meat products and dairy products^(2,3,7,8)^. During absorption in the intestines and subsequent uptake in the organs of animals, MK-4 is the only unsaturated isoprene unit metabolically converted from PK and possibly other MK^(9–11)^.

Vitamin K is predominantly known for its role as a cofactor in the activation of vitamin K-dependent proteins, also known as Gla-proteins, in various tissues^(1,6)^. These Gla proteins are involved in a variety of physiological processes, including blood coagulation, bone mineralisation and the inhibition of vascular calcification and corresponding reduction of cardiovascular risk^(12–15)^. Currently, seventeen different Gla-proteins are recognised, several with yet unknown functions, suggesting that even more vitamin K-dependent functions may be revealed in the future^(12)^. Most of our current understanding of vitamin K pertains to PK. However, several studies suggest that MK may have functions unrelated to PK intake and, for some health effects, may have greater bioactivity than PK^(14–17)^. The uncertainties are acknowledged in the current adequate intake (AI) of 1 µg PK per kg body weight per day for all age and sex groups set by the European Food Safety Authority (EFSA)^(1)^. This AI is exclusively related to PK’s function in haemostasis and has remained unchanged since it was first introduced in 1993 by the former Scientific Committee for Food^(1)^.

Food composition data on vitamin K, and particularly MK, are limited in most countries^(1,3,6,14)^. The MK and PK contents of many foods remain unknown, and the lack of comprehensive food composition data severely limits further research on the associations between vitamin K intake and health^(5,6,17)^. In its 2017 report, EFSA highlighted the need for more extensive and precise analytical data on PK and MK in foods to elucidate their specific biological role and establish corresponding dietary reference values^(1)^. This request was also emphasised in the Nordic Nutrition Recommendations 2023^(18)^. Similarly, in 2018, the Norwegian Scientific Committee for Food and Environment^(19)^ expressed the need to acquire and incorporate data on PK and MK in the Norwegian food composition database, which currently lacks data on vitamin K^(20)^. Existing studies have investigated the contents of PK and MK in foods such as dairy products, particularly cheeses and fermented foods^(7,8,21–23)^. However, the content of vitamin K in many foods remains unknown^(17)^. As a food group, shellfish comprise a diverse range of species and are considered particularly nutrient-dense^(24,25)^. Nevertheless, to our knowledge, their vitamin K content has not been extensively evaluated. The objective of this study was therefore to quantify the contents of PK and MK (MK-4 to MK-10) in various shellfish species. By doing so, we aimed to contribute valuable data and knowledge on vitamin K food composition, thereby enhancing the understanding of vitamin K. We also aimed to assess the potential contribution of PK from shellfish to the AI for adults and explore seasonal differences in the content of vitamin K in blue mussels.

## Methods

### Sampling

A total of 323 samples of shellfish were collected between 2020 and 2023 from Norwegian supermarkets, fish markets, national monitoring programmes, surveys along the coast of Norway and from local fishermen. An overview of the samples, including processing methods and sampling locations, is provided in Table 1, whereas a more detailed overview is provided in the online Supplementary material (Appendix 1). Samples collected from supermarkets were purchased during the autumn of 2020 and 2021 from local supermarkets and fish markets in and around Bergen, Norway. These products reflect the shellfish products available to Norwegian consumers during the autumn of 2020 and 2021, as all shellfish products and product ranges available at the time were purchased for analysis. Three different samples were collected from various batches, per product, per year. Additionally, some samples of bivalves (blue mussels and scallops, *n* 56) were collected through the Norwegian Food Safety Authority’s yearly monitoring programme on contaminants and microorganisms in bivalves in 2023^(26)^. Of these, the blue mussel samples were evaluated in terms of seasonal variations. Moreover, samples were collected using research vessels from various locations along the west coast of Norway in 2021, as part of projects carried out by the Institute of Marine Research (IMR). Samples of crustaceans (shrimps, crabs and lobsters) were collected from local fishermen in 2021 and delivered to the IMR from various locations around the west coast of Norway (as part of other projects at the IMR). The samples were processed to varying degrees, ranging from raw, boiled, steamed or immersed in brine. With the exclusion of bivalves collected from the 2023 monitoring programme, the methodology for sample preparation for all shellfish included in this study has been described previously^(25)^. Briefly, the samples were obtained through convenience sampling, where the samples were weighed and measured prior to homogenisation. Only the edible parts of the shellfish, such as the bivalve meat and the brown meat and claw meat of crustaceans, were used in the analysis. Most shellfish samples were analysed as composite samples consisting of a varying number of individuals; however, some samples of crustaceans were prepared as individual samples (Appendix 1). Bivalves collected from the 2023 monitoring programme were stored at –20°C after preparation, prior to analysis. The remainder of shellfish samples were stored at –80°C prior to analysis.

**Table 1.** Overview of the samples of shellfish included in this study

Shellfish product	Number of analytical samples^*^	Processing	Origin	Species
Blue mussels				
Blue mussels, raw	49	Raw, hand-peeled	Monitoring programme	*Mytilus edulis*
Blue mussels, steamed	8	Steamed, hand-peeled	Supermarkets	*Mytilus edulis*
Blue mussels, in brine	4	In brine, pre-peeled, drained	Supermarkets	*Mytilus edulis*
Blue mussels, pre-packaged	4	Pre-steamed, pre-peeled, frozen	Supermarkets	*Mytilus edulis*
	65			
Scallops				
Deep sea scallop, raw	10	Raw, pre-peeled, pre-packaged frozen	Supermarkets	*Placopecten magellanicus*
Great scallop, raw	7	Raw	Monitoring programme	*Pecten maximus*
	17			
Shrimps				
Northern shrimps, peeled	48	Boiled, hand-peeled, frozen	Supermarkets, Surveys, NAO	*Pandalus borealis*
Northern shrimps, unpeeled	7	Boiled, frozen	Supermarkets, Surveys, NAO	*Pandalus borealis*
Northern shrimps, in brine	57	In brine, pre-boiled, pre-peeled, drained	Supermarkets	*Pandalus borealis/jordani*
	112			
Crabs				
Brown crab, claw meat	18	Boiled, fresh and frozen	Supermarkets, Fishermen	*Cancer pagurus*
Brown crab, brown meat	14	Boiled, fresh and frozen	Supermarkets, Fishermen	*Cancer pagurus*
Stuffed brown crab shells	7	Pre-boiled, frozen	Supermarket	*Cancer pagurus*
Snow crab, leg meat	5	Raw, frozen	Surveys, NAO	*Chionoecetes opilio*
Snow crab, hepatopancreas	5	Raw, frozen	Surveys, NAO	*Chionoecetes opilio*
	50			
Lobsters & Crayfish				
Crayfish, tails	2	Boiled, frozen	Supermarkets	*Procambarus clarkii*
Norway lobster, white meat	3	Raw, frozen	Fishermen	*Nephrops norvegicus*
Norway lobster, white meat	4	Boiled, frozen	Fishermen	*Nephrops norvegicus*
Norway lobster, hepatopancreas	5	Raw, frozen	Fishermen	*Nephrops norvegicus*
American lobster, white meat	6	Pre-boiled, frozen	Supermarkets	*Homarus americanus*
American lobster, hepatopancreas	3	Pre-boiled, frozen	Supermarkets	*Homarus americanus*
European lobster, white meat	20	Raw	Fishermen	*Homarus gammarus*
European lobster, hepatopancreas	10	Raw	Fishermen	*Homarus gammarus*
European lobster, white meat	19	Boiled	Fishermen	*Homarus gammarus*
European lobster, hepatopancreas	8	Boiled	Fishermen	*Homarus gammarus*
	80			

NAO, North Atlantic Ocean.

* Most shellfish products were analysed as composite samples consisting of a varying number of individuals. For a complete overview of the included samples, including the number of individuals per composite sample, see Table A.1, Appendix A.

### Sample preparation: raw bivalves

For blue mussels (*Mytilus edulis*) sampled through the monitoring programme, twenty-five raw individuals comprised one composite sample, whereas for great scallops (*Pecten maximus*), ten raw individuals comprised one composite sample. The samples were rinsed with tap water before weight and length were measured, as described previously by Moxness Reksten *et al.* ^(25)^. The shells were opened by cutting the adductor muscle with a knife, before being placed upright for drainage of water. The blue mussel meat was then manually scraped from the shells using a scalpel. For great scallops, the mantle, gills and digestive gland were removed prior to detaching the muscle from the shell. The bivalve samples were not further processed and were thus analysed in their raw state.

### Analysis of vitamin K contents

#### Analytical quality

The analytical determination of vitamin K was performed at the IMR laboratory in Bergen, Norway, which is accredited according to the ISO/IEC 17025 standard. The methodology is based on a European Standard method for the determination of vitamin K_1_ in foodstuffs by HPLC^(27)^ and determines the content of PK, beta, *γ*-dihydrophylloquinone (dihydro-K_1_) and MK-4 to MK-10, including the separation of *cis*- and *transforms* of all vitamin K vitamers. The vitamin K methodology is not accredited due to the absence of certified reference materials (CRM) and proficiency tests available for any of the MK. However, CRM is analysed at least annually for validation and quality control of PK measurements. Additionally, the determination of PK contents is regularly verified through participation in international proficiency tests. Furthermore, reference materials (analytical standards) and in-house quality control materials are included in each sample run and results are recorded and monitored regularly. All procedures were conducted with minimal daylight exposure due to vitamin K’s sensitivity to light.

#### Analysis of vitamin K contents by HPLC-FLD

The analytical procedure, quality assessments and method performance are described in detail by Jensen *et al.* ^(22)^. Briefly, the contents of PK, dihydro-K_1_ and MK in the samples of shellfish were determined by HPLC coupled to a fluorescence detector (HPLC-FLD). A Dionex Ultimate 3000 HPLC system connected to a FLD-3400RS fluorescence detector (Thermo Fisher Scientific, Waltham, MA, USA) was used. The vitamins were separated on a C30 reversed-phase column (Thermo Fisher Scientific, C30 Accucore column) and electrochemically reduced to hydroquinone before detection (Thermo Fisher Scientific, ESA Coulochem III). Peak integration and analysis were performed using Chromeleon, version 7.2.10 (Thermo Fisher Scientific, Dionex Chromeleon 7 Chromatography Data System). The PK and MK contents were calculated using external calibration (standard curves) based on the concentrations of the nine analytical standards (PK, dihydro-K_1_ and MK4 to MK10).

#### Limits of quantification and measurement uncertainty

The limit of quantification (LOQ) for each vitamer varied according to four conditions in each sample: the amount of weighed sample material, the aliquot of the hexane phase, the volume of the final solvent and the volume injected into the HPLC system^(22)^. An overview of the range of varying LOQ used in this article for each vitamer is provided in Table 2, Appendix B. The measurement uncertainty is 50 % for values LOQ-1 µg/kg, 30 % for values 1–10 µg/kg and 20 % for values from 10 to 3000 µg/kg.

### Contribution of phylloquinone vitamin K contents to dietary reference values

The contents of PK in the shellfish samples were assessed in terms of the AI for adults of 1 µg PK per kg body weight per day, as established by EFSA^(1)^. The reference body weight of 70 kg for 18–79-year-old adults, as described by the EFSA^(1)^, was used. Thus, considering the AI and the reference body weight for adults, the dietary reference value for PK is 70 µg/day. Furthermore, in addition to the functions that are related to MK only, MK also possess the same biological functions as PK^(1)^. Thus, we also present a hypothetical scenario in which the total vitamin K content (vitamin K_1_ + K_2_) contributes to the dietary reference value for vitamin K (using the AI for PK). To ensure a standardised basis for comparing the content of vitamin K^(25)^, a portion size of 100 g was therefore used in the calculations for all shellfish products.

### Comparison of phylloquinone vitamin K and menaquinone vitamin K contents in other foods

To further assess the contents of vitamin K in shellfish, we compared the analytical values in this study with the contents of PK and MK in other foods known to be sources of vitamin K. No food composition databases provide values for individual MK, other than the USA Department of Agriculture’s food composition database^(28)^, which only provides values for MK-4 for a selection of foods. Thus, we compared our results with the analytical data provided by Schurgers and Vermeer^(4)^, which is considered the most comprehensive resource of the content of various MK in foods^(5)^. The paper lists values for PK and MK-4 to MK-10 for six common food categories purchased from local supermarkets in the Netherlands. Among these, we considered a selection of food products recognised to be good dietary sources of vitamin K, such as green, leafy vegetables, fermented foods, dairy products, plant-derived oils and margarines and meats. The number of samples in the study ranged from 4 to 15 for each product, from which we used the provided mean value in our estimations.

### Seasonal variations in the vitamin K content of blue mussels

**Figure 1.**
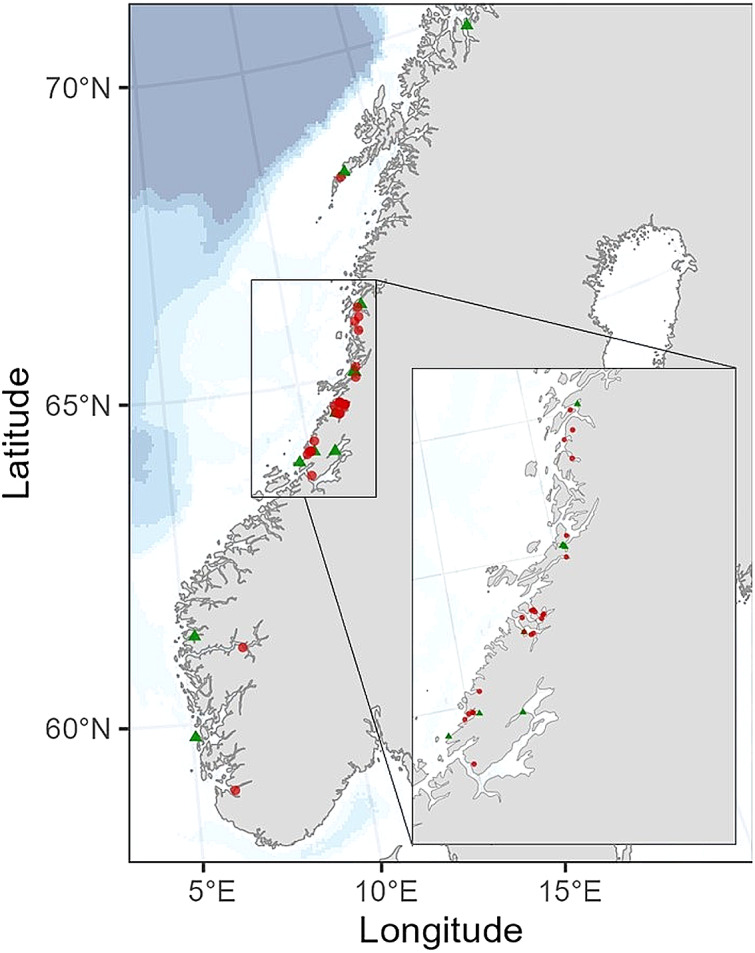
Map illustrating the sampling locations of blue mussels (*Mytilus edulis*) compared for seasonality (green triangles), with twenty-four samples collected twice from the same location, once in spring (*n* 12) and once in late summer (*n* 12). Additionally, other samples of blue mussels (red dots) were used to assess overall monthly variations in vitamin K contents.

The blue mussels sampled from the Norwegian Food Safety Authority’s monitoring programme were evaluated in terms of seasonal variations in vitamin K. This investigation was prompted by unpublished findings from 2016 suggesting variability in vitamin K contents among a subset of blue mussel samples across different seasons of the year. In our study, one composite sample of blue mussels, comprising twenty-five individuals, was sampled from twelve different mussel farming locations along the Norwegian coast in March 2023 (Figure 1). Subsequently, in August and early September 2023, another sample from the same locations was collected again. These samples were categorised into two groups: spring (samples collected in March, *n* 12) and late summer (samples collected in August and early September, *n* 12) and compared with each other. Additionally, blue mussel samples (also containing twenty-five individuals) were collected from various other farming locations along the Norwegian coast in January, March, April, August and October 2023 (*n* 49, Table 2). These samples, in addition to those collected as part of the monitoring programme, were assessed in terms of monthly variations of vitamin K.

**Table 2.** The total number of blue mussels (*Mytilus edulis*) collected and their physical parameters (mean (standard deviation)). The blue mussels that were compared for seasonality are presented in the two last rows

Month	Number of analytical samples^*^	Mean length (cm)		Mean weight (g)	
		Mean	sd	Mean	sd
January	1	7·1^†^		245^†^	
March	15	5·8	1·6	139	41
April	1	7·0^†^		250^†^	
May	1	4·3^†^		56^†^	
August	18	5·8	0·8	131	46
September	2	5·3	1·0	113	65
October	11	5·5	0·6	112	38
Season^‡^					
Spring	12	5·9	0·5	153	38
Late summer	12	6·2	0·6	147	37

* Each sample consisted of twenty-five individual blue mussels pooled together.

† Only one composite sample was collected in January, April and May (*n* 1); thus, the standard deviation (sd) is not presented.

‡ Blue mussels that were compared for seasonality, only included those intentionally sampled twice at the same location; once in spring and once in late summer. Blue mussels sampled for the category ‘spring’ were sampled in March, whereas blue mussels sampled for the category ‘late summer’ were sampled during August and early September.

### Data management, presentation and statistical analyses

Data were registered and extracted from the software Laboratory Information Management System (LIMS), version 8 (LabWare, Wilmington, DE, USA). The vitamin K contents are presented in µg/100 g wet weight, as recommended by the FAO of the United Nations’ guidelines for food composition data^(29)^. The number of significant digits reported, varied according to the measurement uncertainty: for values LOQ-1 µg/100 g, two significant digits were reported; and for values ≥ 1 µg/100 g, three significant digits were reported. To enable the calculation of mean and median values, medium-bound LOQ (LOQ value/2) was applied. Vitamers with > 30 % of the values < LOQ are indicated with a footnote in Table 3. The number of samples with values < LOQ are presented in Table 3, Appendix C. For dihydro-K_1_, values for all shellfish products were < LOQ, and the values for this vitamer are thus not included in the assessment of seasonal variations in the vitamin K content of blue mussels. Statistical analyses were performed using Statistical Package for the Social Sciences (SPSS) for Windows, version 27·0·1·0 (IBM Corporation). The data did not meet the normality assumption (assessed by visual inspection of histograms, QQ plots, and using the Shapiro–Wilk normality test), thus, medians and interquartile ranges (IQR) are presented in the table and text. Mean, standard deviations (sd) and ranges (minimum–maximum) are presented in Table 4, Appendix D. Seasonal differences in the vitamin K content of blue mussels were tested using the non-parametric Mann–Whitney U-test. To test for differences across months, the independent-samples Kruskal–Wallis test was applied, followed by Dunn–Bonferroni s *post hoc* tests for all possible pairwise comparisons. A significance level of *P* ≤ 0·05 was applied. Graphs were created using GraphPad Prism version 8.3.0 for Windows (GraphPad Software, San Diego, CA, USA).

**Table 3.** Contents of phylloquinone, beta, *γ*-dihydrophylloquinone (dihydro K_1_) and menaquinones (MK-4 to MK-10) in various shellfish products. The values are presented as medians and interquartile ranges (IQR), expressed in µg/100 g wet weight^*^

Shellfish product	Phylloquinone		ß, ϒ-2H-K_1_		MK-4		MK-5		MK-6		MK-7		MK-8		MK-9		MK-10		Total
	(K_1_)		(K_1_)^†^		(K_2_)		(K_2_)		(K_2_)		(K_2_)		(K_2_)		(K_2_)		(K_2_)		(K_1_ + K_2_)
Blue mussels																			
Blue mussels, raw	0·44	0·30	0·090	0·00	0·22	0·28	0·090	0·00^‡^	0·39	0·43^‡^	0·14	0·20^‡^	0·090	0·12^‡^	0·12	0·00^‡^	0·21	0·00^‡^	1·79
Blue mussels, steamed	0·58	0·39	0·028	0·034	1·64	2·67	0·11	0·076	1·41	0·810	0·27	0·053	0·26	0·19	0·060	0·039^‡^	0·066	0·079^‡^	4·41
Blue mussels, in brine	0·76	0·14	0·045	0·025	3·86	1·03	0·26	0·048	5·17	4·65	1·14	0·523	0·87	0·23	0·060	0·030^‡^	0·11	0·034^‡^	12·3
Blue mussels, pre-packaged	0·77	0·59	0·045	0·066	4·89	2·29	0·56	0·53	9·17	8·18	2·77	2·27	1·29	1·97	0·060	0·098^‡^	0·11	0·26^‡^	19·7
Scallops																			
Deep sea scallop, raw	0·23	0·15	0·012	0·00	0·098	0·080	0·069	0·083	0·019	0·00^‡^	0·0075	0·00^‡^	0·012	0·00^‡^	0·015	0·00^‡^	0·027	0·00^‡^	0·49
Great scallop, raw	0·023	0·00^‡^	0·023	0·00	0·053	0·026	0·023	0·00^‡^	0·038	0·00^‡^	0·015	0·00^‡^	0·023	0·045^‡^	0·030	0·00^‡^	0·053	0·00^‡^	0·28
Shrimps																			
Northern shrimps, peeled	0·048	0·035	0·012	0·00	0·075	0·081	0·012	0·00^‡^	0·057	0·079^‡^	0·038	0·040	0·012	0·021^‡^	0·015	0·00^‡^	0·027	0·00^‡^	0·29
Northern shrimps, unpeeled	0·40	0·26	0·012	0·00	0·22	0·12	0·18	0·15	1·19	0·77	0·58	0·44	0·34	0·14	0·065	0·12	0·027	0·031^‡^	3·04
Northern shrimps, in brine	0·036	0·012	0·012	0·00	0·039	0·041	0·012	0·00^‡^	0·019	0·00^‡^	0·0080	0·011^‡^	0·012	0·00^‡^	0·015	0·00^‡^	0·027	0·00^‡^	0·18
Crabs																			
Brown crab, claw meat	0·051	0·052	0·012	0·00	0·0075	0·00^‡^	0·012	0·00^‡^	0·019	0·038^‡^	0·011	0·023^‡^	0·012	0·00^‡^	0·015	0·00^‡^	0·027	0·00^‡^	0·17
Brown crab, brown meat	10·7	5·09	0·045	0·0080	0·44	0·40	1·36	2·0	11·9	25·8	6·40	12·3	3·21	4·62	0·81	1·5	0·64	0·89	35·4
Stuffed brown crab shells	3·97	4·62	0·012	0·034	0·11	0·086	0·45	0·35	5·63	6·64	2·80	1·53	1·70	0·39	0·40	0·17	0·24	0·13	15·3
Snow crab, leg meat	0·030	0·028^‡^	0·012	0·00	0·016	0·015^‡^	0·012	0·00^‡^	0·019	0·00^‡^	0·027	0·016	0·012	0·00^‡^	0·015	0·00^‡^	0·027	0·00^‡^	0·17
Snow crab, hepatopancreas	4·61	3·96	0·012	0·00	4·38	2·37	2·51	2·46	59·9	48·9	43·4	35·3	33·3	26·5	13·7	15·1	7·95	9·81	170
Lobsters and Crayfish																			
Crayfish, tails, boiled	0·79^§^		0·012^§^		0·0080^‡^,^§^		0·066^‡^,^§^		0·11^‡^,^§^		0·089^§^		0·057^§^		0·015^‡^,^§^		0·027^‡^,^§^		1·17
Norway lobster, white meat, raw	0·012	0·027^‡^	0·012	0·00	0·47	0·60^‡^	0·012	0·00^‡^	0·019	0·00^‡^	0·0075	0·060^‡^	0·012	0·00^‡^	0·015	0·00^‡^	0·027	0·00^‡^	0·58
Norway lobster, white meat, boiled	0·023	0·012^‡^	0·012	0·0080	0·0075	0·0060^‡^	0·012	0·0080^‡^	0·019	0·014^‡^	0·016	0·0030	0·017	0·014^‡^	0·015	0·011^‡^	0·027	0·020^‡^	0·15
Norway lobster, hepatopancreas, raw	0·54	0·89	0·023	0·00	0·015	0·63^‡^	1·07	0·23	2·32	3·39	1·35	2·80	0·90	0·95	0·21	0·19	0·053	0·00^‡^	7·74
American lobster, white meat, boiled	0·081	0·24^‡^	0·012	0·0030	0·028	0·053^‡^	0·046	0·12^‡^	0·070	0·13^‡^	0·036	0·050^‡^	0·019	0·047^‡^	0·015	0·011^‡^	0·027	0·020^‡^	0·33
American lobster, hepatopancreas, boiled	0·25	5·8	0·023	0·034	0·16	0·30	1·41	2·39	1·33	0·48	0·48	0·18	0·43	0·22	0·030	0·045^‡^	0·11	0·079^‡^	4·22
European lobster, white meat, raw	0·049	0·017	0·012	0·00	0·032	0·026	0·012	0·00^‡^	0·052	0·079^‡^	0·12	0·49	1·21	7·1^‡^	0·015	0·059^‡^	0·027	0·00^‡^	1·52
European lobster, hepatopancreas, raw	0·46	0·30	0·012	0·00	0·021	0·015	0·13	0·057	0·82	1·0	0·55	1·3	1·66	4·09	0·091	0·10	0·027	0·00^‡^	3·75
European lobster, white meat, boiled	0·043	0·044	0·012	0·00	0·024	0·033	0·012	0·00^‡^	0·026	0·063^‡^	0·030	0·038	0·012	0·023^‡^	0·015	0·00^‡^	0·027	0·00^‡^	0·20
European lobster, hepatopancreas, boiled	0·38	0·25	0·012	0·00	0·018	0·018	0·12	0·12^‡^	0·45	0·58	0·30	0·18	0·24	0·13^‡^	0·15	0·054^‡^	0·027	0·00^‡^	1·55

MK, menaquinone.

* For individual values below the limit of quantification (LOQ), the LOQ value was divided by two (medium-bound LOQ: LOQ/2) to enable the calculation of the median and interquartile range (IQR).

† All values for all shellfish products were below the LOQ for ß, ϒ-2H-K_1_. The IQR varied due to differences in the LOQ, caused by variations in the analytical conditions (see Table 2, Appendix B).

‡ This product consists of values where over 30 % of the values fall below the LOQ. For more information on the number and percentage of the samples that presented values below the LOQ for each vitamer, see Table 3, Appendix C.

§ The IQR could not be calculated due to a low number of samples (*n* 2).

## Results

### Phylloquinone, dihydro-K_1_ and total vitamin K contents in shellfish

Table 3 shows the mean contents of PK, dihydro-K_1_ and total vitamin K (K_1_ + K_2_) measured in the 323 shellfish samples. The median contents of PK varied considerably between each shellfish category, between shellfish products and between samples of the same shellfish products. The white meat of raw Norway lobster contained the lowest amount of PK (0·023 µg/100 g), followed by boiled Norway lobster (white meat) and great scallops, of which a high percentage of values were below LOQ (67 and 100 %, respectively). Overall, crabs as a group contained the highest contents of PK, albeit with considerable variations within the group (Table 4, Appendix D). Specifically, the brown meat of brown crab displayed the highest median content of PK at 10·7 µg/100 g; however, substantial variations were noted, ranging from 3·3 to 17 µg/100 g. It was further evident that the content of PK was considerably higher in the brown meat and hepatopancreas of crustaceans than in their white meat counterparts. For dihydro-K_1_, values for all shellfish products were below the LOQ (Table 3, Appendix C). The total vitamin K content varied from 0·15 µg/100 g in the white meat of boiled Norway lobster to 170 µg/100 g in the hepatopancreas of snow crab. For blue mussels as a group, the total vitamin K content was lowest in raw samples (1·79 µg/100 g), higher in steamed samples (4·41 µg/100 g), further increased in blue mussels in brine (12·3 µg/100 g) and the highest in pre-packaged blue mussels (19·7 µg/100 g). Furthermore, the total vitamin K content was also considerably higher in the brown meat and hepatopancreas of most crustaceans than in their white meat counterparts. As an example, the median vitamin K content in the hepatopancreas of snow crab (170 µg/100 g) was 1000-fold higher than the median content in the legs (0·17 µg/100 g).

### Menaquinone contents in shellfish

Table 3 presents the measured contents of MK-4 to MK-10 in the shellfish products. The overall dominating MK was MK-6, followed by MK-4. The hepatopancreas of snow crab exhibited the highest contents of all MK, except for MK-4, which was highest in pre-packaged blue mussels. For MK-9 and MK-10, nearly all samples (81 and 91 %, respectively) had values below the LOQ (online Supplementary Table S3, Appendix C). A high number of samples below the LOQ was also evident for MK-5 (72 %). Raw samples of European lobster (both white meat and hepatopancreas) were the only shellfish products to exhibit MK-8 as the most abundant MK. These elevated levels of MK-8 were however not observed in the boiled samples of European lobster, where MK-6 was the most abundant MK. In blue mussels, MK-6 was the most abundant MK for all products regardless of processing, except for steamed blue mussels, where MK-4 was the most abundant. For scallops, nearly all vitamers, except for MK-4, had a high share of values below the LOQ (70–100 %). Similarly, for shrimps, the majority of values for the various MK were below the LOQ, except for MK-4 in shrimps in brine and both MK-4 and MK-7 in peeled shrimps. In unpeeled shrimps, the MK content was substantially higher than the MK content in peeled shrimps and shrimps in brine, with MK-6 as the most abundant MK (1·19 µg/100 g). The MK content of brown meat and the hepatopancreas of crustaceans was substantially higher than the PK content and compared with the other shellfish products. These matrices presented considerably higher levels of long-chain MK. It was also evident that the hepatopancreas of snow crab and the brown meat of brown crab had particularly high contents of long-chain MK (especially MK-6 to MK-9). The standard deviations were large for several of the samples, particularly for samples presenting high contents of MK, such as blue mussels and crustaceans, indicating large inter-individual variations (Table 4, Appendix D).

### Contribution to adequate intake

**Figure 2.**
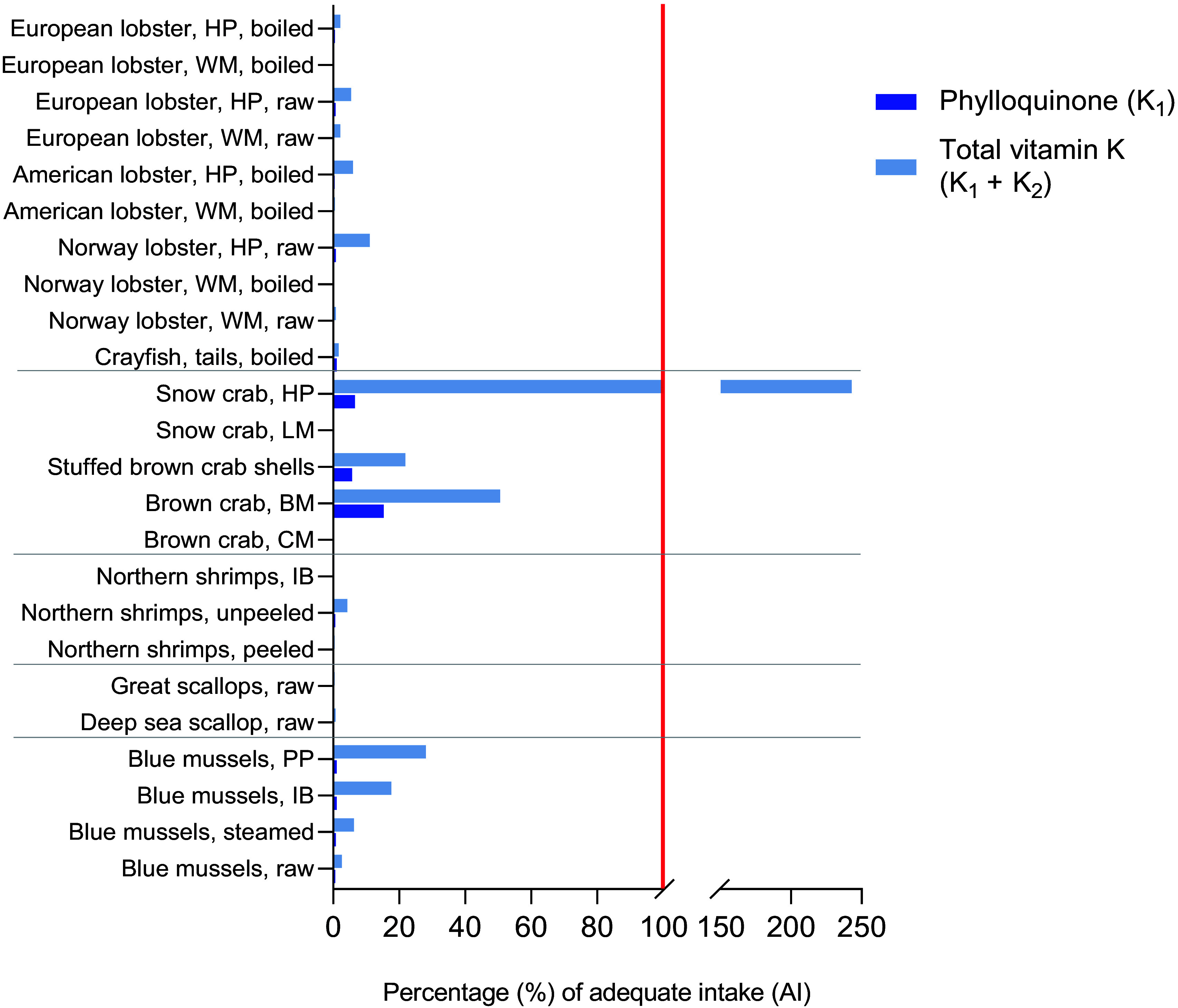
The estimated contribution of a 100 g serving of the shellfish products (median contents) to the adequate intake (AI) of phylloquinone is presented in dark blue. The red line indicates 100 % of the AI. A hypothetical scenario where the total vitamin K content (vitamin K_1_ + vitamin K_2_) is compared against the AI of phylloquinone is presented in light blue. BM, brown meat; CM, claw meat; HP, hepatopancreas; IB, in brine; LM, leg meat; PP, pre-packaged; WM, white meat.

When comparing the mean contents of PK in the shellfish product to the AI for PK, we found that intake of 100 g of all shellfish products, with the exception of the brown meat of brown crab and hepatopancreas of snow crab, resulted in meeting ≤ 1 % of the AI for PK (Figure 2). Whereas the brown meat of brown crab was estimated to contribute approximately 15 % of the AI, 100 g of the hepatopancreas of snow crab was able to contribute approximately 7 % of the AI. However, when evaluating the total vitamin K content’s hypothetical contribution to the dietary reference value (using the AI for PK), several shellfish products were able to meet ≥ 15 % of the AI due to their high contents of MK. The hepatopancreas of snow crab, for example, was estimated to meet 243 % of the AI when considering both PK and MK contents, whereas the brown meat of brown crab was estimated to meet 51 % of the AI.

### Comparison with other foods

**Figure 3.**
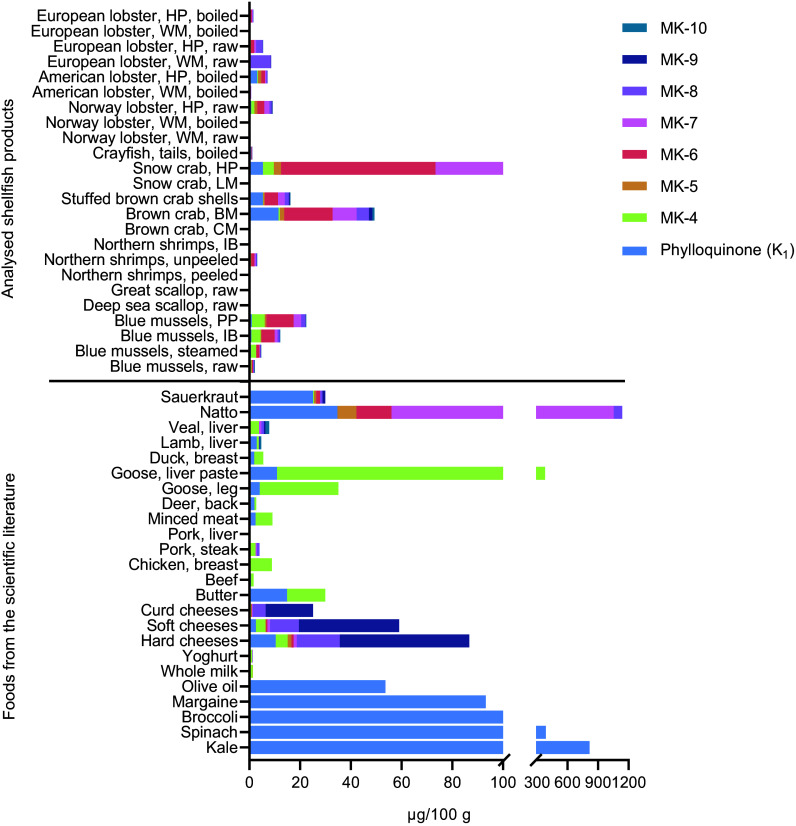
Comparison of the mean content of vitamin K in different shellfish and other foods recognised as good sources of vitamin K. The analytical mean values of other foods were obtained from Schurgers and Vermeer^(4)^. BM, brown meat; CM, claw meat; HP, hepatopancreas; IB, in brine; LM, leg meat; PP, pre-packaged; WM, white meat.

The analysed contents of PK and MK-4 to MK-10 in the shellfish included in this study were compared with those of other foods recognised to be high in vitamin K (Figure 3). Several of the shellfish samples included in this study contained vitamin K contents comparable to, or exceeding, foods recognised as good sources of vitamin K. For instance, the hepatopancreas of snow crab (total vitamin K: 170 µg/100 g) was surpassed only by natto (1138 µg/100 g), goose liver paste (380 µg/100 g), kale (817 µg/100 g) and spinach (387 µg/100 g). The particularly high content of MK-6 observed in the hepatopancreas of snow crab (59·9 µg/100 g) exceeded that of any foods previously reported in the literature. Furthermore, many of the analysed shellfish products exceeded the total vitamin K content of the majority of meat products, such as beef, chicken breast, pork steak and liver, deer, duck, lamb and veal, as well as dairy products such as milk and yoghurt. Conversely, many of the shellfish products, such as scallops, peeled shrimps and shrimps in brine, the claw meat and leg meat of crabs and the white meat of most lobster species, did not surpass the majority of foods recognised as good sources of vitamin K.

### Seasonal variations of vitamin K content of blue mussels

**Figure 4.**
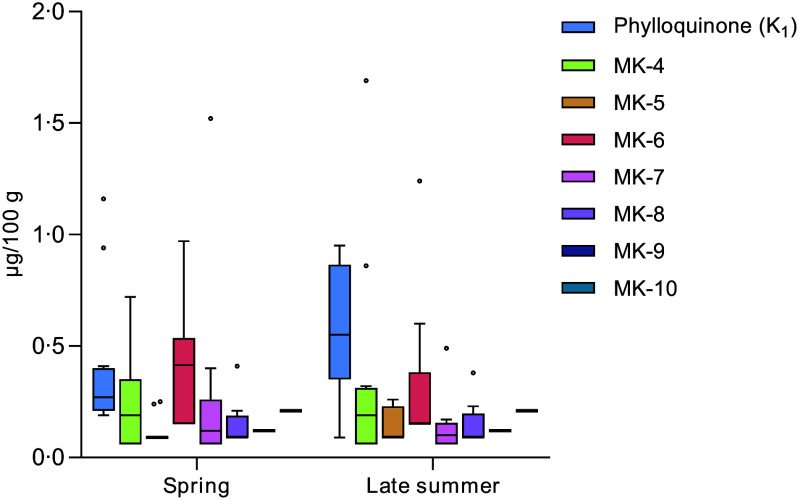
Seasonal differences between the blue mussels (*Mytilus edulis*) sampled during spring *v*. late summer. Each box represents the interquartile range (IQR), the median is denoted by the black line within the box, the whiskers denote variability (values within 1·5 IQR) and dots denote observations outside the range of 1·5 IQR.

The seasonal variations in vitamin K between raw blue mussels sampled at the same geographical locations during spring and late summer, respectively, are presented in Figure 4. No significant differences (*P* > 0·05) were observed for any of the vitamers. The largest differences were found for the median contents of PK (0·27 µg/100 g in spring and 0·55 µg/100 g in late summer) and MK-6 (0·42 µg/100 g in spring and 0·15 µg/100 g in late summer) although not significant. When evaluating the differences in total vitamin K contents (vitamin K_1_ + K_2_), no significant differences were found either. Furthermore, the samples were evaluated in terms of geographical location (North/South), revealing no significant differences in vitamin K contents between samples collected in the Northern *v*. Southern regions of Norway.

**Figure 5.**
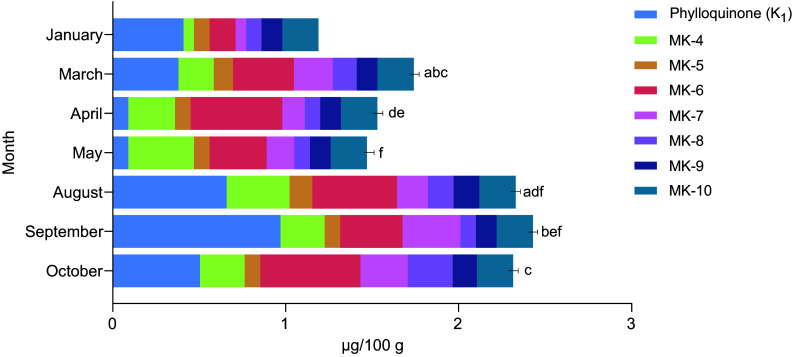
The contents of phylloquinone (PK) and menaquinones (MK-4 to MK-10) in blue mussels (*Mytilus edulis*) sampled during various months of the year. Statistically significant differences (*P* < 0·05) for PK are indicated using letters. If two or more bars are marked with the same letters, significant differences were found between these months.

All raw blue mussels sampled in 2023 were also compared in terms of monthly variations (Figure 5). An increasing trend was observed, indicating an increase in vitamin K content throughout the year, with higher levels recorded in the autumn compared with the spring. The total vitamin K content of the mussels sampled in the months of January–May was also found to be significantly lower (*P* = 0·003) than that of the blue mussels sampled in August–October. In terms of monthly variations, significant differences were observed in the PK content between several of the months, as indicated in Figure 5. For MK, differences were observed between August and October for MK-5 (*P* = 0·042), March and October for MK-7 (*P* = 0·043) and March (*P* = 0·003), August (*P* = 0·007) and September (*P* = 0·025) in comparison to October for MK-8.

## Discussion

This study provides novel analytical insights into the vitamin K content of shellfish, a food group not previously investigated. Thus, our study contributes to the limited food composition data on vitamin K worldwide, particularly in Norway. Furthermore, whereas existing scientific literature typically reports the content of PK and only one or two MK, our study provides analytical data on PK, dihydro-K_1_ and MK-4 to MK-10 in more than 300 samples. The total vitamin K content was generally < 10 µg/100 g for most shellfish products, with the exception of blue mussels in brine and pre-packaged blue mussels, the brown meat of brown crab, stuffed brown crab shells and the hepatopancreas of snow crab. Among these, the hepatopancreas of snow crab displayed the clearly highest content of total vitamin K (170 µg/100 g), followed by the brown meat of brown crab (35·4 µg/100 g). When comparing our results with foods recognised as good sources of vitamin K^(4)^, many of the shellfish products exceeded the total vitamin K contents found in most meat products and some dairy products. Overall, the hepatopancreas of crustaceans, the brown meat of brown crab (including stuffed crab shells) and blue mussels may particularly be considered good sources of vitamin K when compared with other foods acknowledged for their vitamin K content. The predominant MK detected in most shellfish products was MK-6, followed by MK-4. The hepatopancreas of snow crab exhibited a notably high content of MK-6, a feature not observed in any of the foods already acknowledged as good sources of vitamin K. Moreover, as dihydro-K_1_ is formed during the hydrogenation of plant oils, which is an industrial process^(30)^, it is not surprising that all samples of shellfish had dihydro-K_1_ contents below the LOQ. Furthermore, no significant differences in the content of any vitamin K vitamer were observed between blue mussels sampled during spring *v*. blue mussels sampled during late summer. Conversely, significant monthly variations were observed from January through October, with a trend towards higher contents in the autumn months.

To the best of our knowledge, previous studies have not extensively investigated the vitamin K contents in shellfish. In the study by Schurgers and Vermeer^(4)^, which assessed the vitamin K contents in various foods, only seven samples of ‘prawn’ (without further specification) were analysed. In that study, the mean content of PK was determined to be 0·1 µg/100 g, whereas the contents of all MK were reported as ‘not detectable’. Although comparison is difficult due to lack of documentation on sampling and sample preparation, these findings align with the results presented in our study, where the total mean content of vitamin K in peeled shrimps and shrimps in brine was found to be low (< 0·1 µg/100 g). Similarly, in a study by Elder *et al.* ^(31)^, which analysed the contents of PK, dihydro-K_1_ and MK-4 in a range of foods in the USA, low vitamin K contents were reported in one sample (*n* 1) of cooked and canned shrimps (MK-4:0·2 µg/100 g, not detectable contents of PK and dihydro-K_1_). Additionally, one sample of canned crab (*n* 1) was analysed in the same study, yielding non-detectable contents of all three analytes. Moreover, although several other studies have analysed the contents of vitamin K across a diverse range of various food groups^(3,23,32–35)^, shellfish have not been previously examined. Despite limited investigation, in other seafood such as fish, the content of vitamin K_2_ is considered generally low (but detectable), and MK other than MK-4 have only been identified in a small number of fish species^(17,36–38)^.

### White meat *v*. hepatopancreas of crustaceans

Our results revealed considerably higher contents of vitamin K in the brown meat and hepatopancreas of crustaceans than in their white meat counterparts. In a previous study, the MK content was found to vary by the percentage of fat present in dairy products^(23)^, with a significantly higher content of MK in full-fat products than in dairy products with a reduced fat content. Higher contents of vitamin K in fatty fish than in lean fish have also been reported^(38)^. The fat content in the claw meat and leg meat (white meat) of brown crab and snow crab is substantially lower than in the brown meat and hepatopancreas, with values of 1·1 *v*. 9·8 g/100 g for brown crab, and 1·3 *v*. 24 g/100 g for snow crab, respectively^(25)^. As a fat-soluble vitamin, it is not surprising that the highest contents of vitamin K were found in tissues with the highest fat content. However, despite having similarly high-fat contents as the hepatopancreas of brown crab and snow crab, elevated vitamin K contents were not observed in the hepatopancreas of Norway lobster, American lobster or raw and boiled European lobster, with fat contents of 9·3, 11, 23 and 25 g fat/100 g, respectively^(25)^. This implies that additional factors, such as diet, environmental conditions or other external or internal influences, may affect the content of vitamin K in shellfish. In terrestrial animals, MK and particularly long-chained MK, have previously been reported in the liver^(4,31,37)^, which is thought to be the largest storage organ of vitamin K^(1,39)^. This may also partly explain the high contents of MK-6 to MK-9 found in the brown meat of brown crab and in the hepatopancreas of snow crab, as both of these tissues comprise the liver. Considerably higher contents of MK-4 have also previously been reported in goose liver paste (370 µg/100 g) compared with goose leg (31·0 µg/100 g)^(4)^. Moreover, given that many of the shellfish included in this study have a diet rich in phytoplankton^(40–42)^, the dominating prevalence of MK-4 in shellfish such as blue mussels, scallops and shrimps could potentially be a result of the conversion of PK (or other MK) to MK-4. For instance, for deep sea scallops, nearly all values were below the LOQ, except for values for PK and MK-4, where none were below the LOQ. Although there is limited data on the vitamin K content in phytoplankton, certain algae have been reported to contain very high contents of PK^(22,32,43)^. Whereas the conversion of menadione in fish feed to MK-4 has been reported in various fish species^(44)^, this has not yet been documented in shellfish, indicating a need for further research to clarify this aspect.

### Variations in vitamin K contents

For several of the shellfish products analysed in this study, considerable intra-species variation was observed. For instance, in the hepatopancreas of snow crab, the content of MK-6 varied from 23·4 to 95·6 µg/100 g, whereas the content of MK-7 varied from 19·6 to 70·8 µg/100 g. Large variations were also evident in products with relatively low median total vitamin K contents, such as the white meat of raw European lobster, where the content of MK-8 ranged from 0·012 (< LOQ) to 56·0 µg/100 g. The median value for raw white meat of European lobster is based on twenty composite samples, with ten samples collected in Hvaler (Eastern Norway) and the other ten in Austevoll (Western Norway). In lobsters sampled from Eastern Norway, the range for MK-8 varied from 2·4 to 56 µg/100 g, whereas for those sampled from Western Norway, all values were below the LOQ. All samples were collected in November, suggesting that factors other than season, such as geographic location, differences in diet or other unknown factors might account for the observed variability. No substantial differences were observed in the contents of PK or the other MK between the samples. Moreover, large variations have been observed in various food products from terrestrial animals, where the country of origin and regional disparities have been found to affect the content of vitamin K^(5)^. While these differences have previously been attributed to the use of menadione in animal feeds and disparities in food production, such factors cannot account for the variations observed in the shellfish products analysed in this study.

### Seasonal variations in vitamin K contents of blue mussels

When examining seasonal differences between blue mussels harvested at the same geographic location, we found no significant differences between spring and late summer. However, when evaluating monthly variations across the year, significant differences were observed for several of the vitamers, with a clear trend of higher vitamin K contents in the autumn months, particularly in September. As blue mussels are suspension feeders and thus primarily feed on phytoplankton present in the surrounding water^(40,42)^, the onset of seasonal algal blooms is one plausible explanation for this. Along the Norwegian coast, algal blooms typically occur from spring to late summer^(45)^. As food availability primarily controls the growth of filter-feeding bivalves^(42,46)^, the increased availability of algae in the water during early summer months may result in blue mussels accumulating more nutrients, including vitamin K, towards late summer and early fall compared with preceding months. For example, the blue mussel sample analysed in May (*n* 1) was considerably smaller in size and lighter in weight than the samples collected in all other months. It should, however, also be noted that the number of samples varied considerably between the different months (from 1–18 samples), resulting in a less-than-optimal setup for evaluating monthly variations. Additionally, samples were not collected from all months of the year. Another limitation is the storage temperature of –20°C applied to all blue mussels assessed for seasonal variations, which may have impacted the vitamin K contents of the samples. Significantly lower PK and MK-4 contents have previously been reported in samples of plaice – which have a similar low-fat content to blue mussels (< 3 g/100 g^(25)^) – when stored at –20°C, as opposed to –80°C^(47)^. Furthermore, the disparities in vitamin K contents may also be attributed to geographical variances, a factor not comprehensively assessed in our study, given that the majority of samples were collected in close proximity to each other within the same geographic area of mid-Norway. Consequently, further research should be conducted on the impact of seasonal and geographic variations on the content of vitamin K in blue mussels, and in other shellfish.

### Degree of processing

We further observed a trend of higher contents of vitamin K in steamed blue mussels than in raw blue mussels, with an even higher content observed in blue mussels in brine, and, ultimately, pre-packaged blue mussels. The experimental design of this study does not facilitate an exact assessment of the effect of processing on the content of vitamin K, given that identical samples were not analysed across all categories. Nonetheless, some hypotheses can be proposed. The observed increase in vitamin K contents with the degree of processing may be attributed to several factors. In general, cooking (boiling/steaming) may reduce the water content in foods^(29)^, which may lead to an increase in the content of vitamin K per 100 g sample, as the relative proportion of nutrients in the food becomes more concentrated. For the samples used in this study, the dry matter/water content of all blue mussel samples has been analysed previously^(25)^; however, no clear trend could be observed. As expected, the median water content was highest in raw samples (81·8 g/100 g) and decreased in steamed blue mussels (76·2 g/100 g). For blue mussels in brine, the water content was higher than steamed samples (79·3 g/100 g), likely attributed to the presence of brine water surrounding the mussels. In pre-packaged blue mussels (78·6 g/100 g), however, the water content was also increased, posing a challenge in correlating the high content of vitamin K with the decrease in water content. Limited data regarding the impact of cooking or processing on the content of MK in foods is available in the scientific literature; however, some studies have investigated the effect of cooking on the content of PK in various vegetables^(48,49)^. A general trend towards higher contents of PK in cooked compared with raw vegetables was found in both studies, although this was not consistent across all food comparisons.

### Contribution to adequate intakes

Very few of the shellfish products were found to significantly contribute to the AI of PK. The brown meat of brown crab presented the highest contribution, of which a 100 g serving was estimated to provide 15 % of the AI of PK. Despite their relatively low PK content, many shellfish products contained high amounts of MK. In a hypothetical scenario, this resulted in an estimated potential contribution of several shellfish products to the AI ranging from 15 to 243 % when accounting for the presence of MK. As both forms of vitamin K activate vitamin K-dependent proteins^(1,6,15)^, this hypothetical scenario consisted of simply combining the contents of PK and MK and comparing them to the established AI for PK. However, it is important to note that this scenario has limited value, as the current AI is based solely on the nutritional requirements for PK and does not incorporate MK. Moreover, it has been suggested that higher recommendations for vitamin K intake should be established, as higher dietary vitamin K intakes are required for adequate carboxylation of extrahepatic Gla proteins, than what is necessary to maintain coagulation^(12,15)^. Additionally, the need for establishing a dietary reference value for MK separate from PK, contending that the beneficial effects of vitamin K_2_ are not adequately covered by the current AI, has been argued^(16)^. This is also in relation to the absorption, half-life and carboxylation efficiency of vitamin K_2_ in relation to vitamin K_1_. Furthermore, the brown meat of brown crab and the hepatopancreas of other crustaceans should be noted as potential sources of contaminants. For brown crab in particular, high contents of cadmium have been reported^(50)^. Therefore, despite the high contents of vitamin K, a cautionary approach should be applied regarding the consumption of brown meat and hepatopancreas. For vulnerable groups such as pregnant women and children, caution is particularly warranted.

### Conclusions

The analytical contents of PK, dihydro-K_1_ and MK-4 to MK-10 in a range of various shellfish species and tissues of varying degrees of processing are described in this paper. Despite considerable variations, the majority of shellfish products contained low contents of total vitamin K (< 10 µg/100 g). High contents of vitamin K were found in the hepatopancreas of snow crab, the brown meat of brown crab, stuffed brown crab shells, pre-packaged blue mussels and blue mussels in brine. In general, the hepatopancreas of crustaceans contained considerably higher contents of vitamin K than their white meat counterparts. Furthermore, compared with other foods recognised as sources of vitamin K, such as meat and dairy products, many of the shellfish products exceeded the contents of total vitamin K found in these foods. This was particularly attributed to a high content of MK, as most shellfish products contained little PK, as highlighted by the insignificant contribution to the AI for adults. Furthermore, the content of vitamin K in blue mussels did not significantly differ between spring and early summer. To the best of our knowledge, this is the first study where shellfish have been extensively analysed for their contents of vitamin K, and as the majority of shellfish species analysed in this study are prevalent and commonly consumed in several regions of the world, these data provide an important contribution to the limited food composition data on vitamin K worldwide. However, we observed considerable variability in vitamin K contents both among and within shellfish species, which may impact the external validity of our results. Consequently, more research should be conducted to explore the substantial variations in the vitamin K contents of shellfish, which may be affected by factors such as geography, season and diet.

## Supporting information

Moxness Reksten et al. supplementary material 1 Moxness Reksten et al. supplementary material

Moxness Reksten et al. supplementary material 2 Moxness Reksten et al. supplementary material

Moxness Reksten et al. supplementary material 3 Moxness Reksten et al. supplementary material

Moxness Reksten et al. supplementary material 4 Moxness Reksten et al. supplementary material

## Supplementary material

For supplementary material/s referred to in this article, please visit https://doi.org/10.1017/S0007114525000261
