# Supplementary material for: Quantification of vitamin K (phylloquinone and menaquinones 4–10) in various shellfish
Source: Br J Nutr. 2025 Feb 13;133(4):469–80. doi: 10.1017/S0007114525000261 (PMC12011545; doi:10.1017/S0007114525000261)
Supplement: Moxness Reksten et al. supplementary material 1 — Moxness Reksten et al. supplementary material [file S0007114525000261sup001.docx]

| **Product**  **name and brand** | **Categorised as ^a^** | **Sampling method** | **Sampling year** | **Batch number** | **Expiration date**  (dd.mm.yy) | **Origin^b^** | **Species** | **Physical parameters^c^** | |
| --- | --- | --- | --- | --- | --- | --- | --- | --- | --- |
|  |  |  |  |  |  |  |  | **Weight**  **(g)** | **Length (cm)** |
| **Blue mussels,** raw (49 composite samples) | Blue mussels, raw | NFSA | 2023 | Sampled: February-October | - | Along the Norwegian coastline (‘Blåskjellvarselet’) | *Mytilus edulis* | 132 ± 52 | 5.7 ± 0.8 |
| **Blue mussels**, 1 kg bag, «Norgeskjell» | Blue mussels, steamed | Super-market | 2020 | 124 (Produced: 13.10.20) | 25.10.20 | Trøndelag, Norway | *Mytilus edulis* | 211 | 5.5 ± 0.9 |
| **Blue mussels**, 1 kg bag, «Norgeskjell» | Blue mussels, steamed | Super-market | 2021 | T1  (Produced: 01.09.21) | 11.09.21 | Trøndelag, Norway | *Mytilus edulis* | 241 | 5.0 ± 0.7 |
| **Blue mussels**, 1 kg bag, «Norgeskjell» | Blue mussels, steamed | Super-market | 2020 | 125 (Produced: 14.10.20) | 26.10.20 | Trøndelag, Norway | *Mytilus edulis* | 228 | 5.2 ± 0.7 |
| **Blue mussels**, 1 kg bag, «Norgeskjell» | Blue mussels, steamed | Super-market | 2021 | T3  (Produced: 06.09.21) | 16.09.21 | Trøndelag, Norway | *Mytilus edulis* | 248 | 5.3 ± 0.6 |
| **Blue mussels**, 1 kg bag, «Norgeskjell» | Blue mussels, steamed | Super-market | 2020 | 123 (Produced: 12.10.20) | 24.10.20 | Trøndelag, Norway | *Mytilus edulis* | 237 | 5.2 ± 0.7 |
| **Blue mussels**, 1 kg bag, «Norgeskjell» | Blue mussels, steamed | Super-market | 2021 | 139 (Produced: 02.09.21) | 13.09.21 | Trøndelag, Norway | *Mytilus edulis* | 279 | 5.0 ± 0.5 |
| **Blue mussels**, 1 kg bag, «Norgeskjell» | Blue mussels, steamed | Super-market | 2020 | 126 (Produced: 15.10.20) | 27.10.20 | Trøndelag, Norway | *Mytilus edulis* | 208 | 5.9 ± 0.8 |
| **Blue mussels**, 1 kg bag, «Norgeskjell» | Blue mussels, steamed | Super-market | 2021 | 142 (Produced: 06.09.21) | 18.09.21 | Trøndelag, Norway | *Mytilus edulis* | 300 | 4.8 ± 0.5 |
| **Blue mussels**, in brine, “Fiskemannen” | Blue mussels, in brine | Super-market | 2020 | 22:27, L4 | 26.10.20 | Denmark | *Mytilus edulis* | 205 | - |
| **Blue mussels**, in brine, “Fiskemannen” | Blue mussels, in brine | Super-market | 2021 | 14:28, L4 | 04.10.21 | Denmark | *Mytilus edulis* | 215 | - |
| **Blue mussels**, in brine, “Fiskemannen” | Blue mussels, in brine | Super-market | 2021 | 13:46, L4 | 15.11.21 | Denmark | *Mytilus edulis* | 178 | - |
| **Blue mussels**, in brine, “Fiskemannen” | Blue mussels, in brine | Super-market | 2021 | 22.57, L4 | 29.11.21 | Denmark | *Mytilus edulis* | 205 | - |
| **Blue mussels**, pre-peeled, pre-steamed, frozen, “Fiskemannen” | Blue mussels, pre-packaged | Super-market | 2020 | 143 | 22.05.21 | FAO 27 | *Mytilus edulis* | 221 | - |
| **Blue mussels**, pre-peeled, pre-steamed, frozen, “Fiskemannen” | Blue mussels, pre-packaged | Super-market | 2020 | 128 | 07.05.21 | FAO 27 | *Mytilus edulis* | 220 | - |
| **Blue mussels**, pre-peeled, pre-steamed, frozen, “Fiskemannen” | Blue mussels, pre-packaged | Super-market | 2021 | 085 | 26.03.22 | FAO 27 | *Mytilus edulis* | 221 | - |
| **Blue mussels**, pre-peeled, pre-steamed, frozen, “Fiskemannen” | Blue mussels, pre-packaged | Super-market | 2021 | 131 | 11.05.22 | FAO 27 | *Mytilus edulis* | 219 | - |
| **SCALLOPS** |  |  |  |  |  |  |  |  |  |
| **Scallops**, pre-peeled, frozen, «Fiskeriet” | Deep sea scallop, raw | Super-market | 2021 | 12144 (Produced: 04.09.20) | 04.09.22 | FAO 21 | *Placopecten magellanicus* | 407 | - |
| **Scallops**, pre-peeled, frozen, «Fiskeriet” | Deep sea scallop, raw | Super-market | 2021 | 12292 (Produced: 04.09.20) | 04.09.22 | FAO 21 | *Placopecten magellanicus* | 419 | - |
| **Scallops**, pre-peeled, frozen, «Fiskeriet” | Deep sea scallop, raw | Super-market | 2020 | 11464 (Produced: 12.02.20) | 12.02.22 | FAO 21 | *Placopecten magellanicus* | 376 | - |
| **Scallops**, pre-peeled, frozen, “Polar Seafood Greenland” | Deep sea scallop, raw | Super-market | 2020 | 935356 (Produced: 19.12.19) | 19.12.21 | FAO 21 | *Placopecten magellanicus* | 440 | - |
| **Scallops**, pre-peeled, frozen, “Polar Seafood Greenland” | Deep sea scallop, raw | Super-market | 2021 | 90313 (Produced: 01.06.21) | 01.06.23 | FAO 21 | *Placopecten magellanicus* | 507 | - |
| **Scallops**, pre-peeled, frozen, “Fiskemannen” | Deep sea scallop, raw | Super-market | 2021 | 2011047 (Produced: 04.03.21) | 01.09.22 | FAO 21 | *Placopecten magellanicus* | 332 | - |
| **Scallops**, pre-peeled, frozen, “Fiskemannen” | Deep sea scallop, raw | Super-market | 2021 | 2001125 (Produced: 01.07.21) | 22.08.22 | FAO 21 | *Placopecten magellanicus* | 295 | - |
| **Scallops**, pre-peeled, frozen, “Øfas” | Deep sea scallop, raw | Super-market | 2020 | NOR_11303 (Produced: 25.07.19) | 25.07.21 | FAO 21 | *Placopecten magellanicus* | 423 | - |
| **Scallops**, pre-peeled, frozen, “Øfas” | Deep sea scallop, raw | Super-market | 2020 | NOR_11503 (Produced: 07.10.19) | 07.10.21 | FAO 21 | *Placopecten magellanicus* | 423 | - |
| **Scallops**, pre-peeled, frozen, “Øfas” | Deep sea scallop, raw | Super-market | 2021 | NOR_12256 (Produced: 04.09.20) | 04.09.22 | FAO 21 | *Placopecten magellanicus* | 419 | - |
| **Scallops,** raw (7 composite samples) | Great scallop, raw | NFSA | 2023 | Sampled: February-October | - | Along the Norwegian coastline (‘Blåskjellvarselet’) | *Pecten maximus* | 237 ± 101 | 10.5 ± 1.2 |
| **CRABS** |  |  |  |  |  |  |  |  |  |
| **Brown crab, claws and brown meat**, fresh, pre-boiled, 9 individuals (= 3 composite samples) | Brown crab, claw meat  Brown crab, brown meat | Fish market | 2020 | Purchased: 20.10.20 | - | Hitra, Norway | *Cancer pagurus* | 454 ± 108 | Width: 15.0 ± 0.8 Length: 9.5 ± 0.6 |
| **Brown crab, claws and brown meat**, fresh, pre-boiled, 9 individuals (= 3 composite samples) | Brown crab, claw meat  Brown crab, brown meat | Fish market | 2021 | Purchased: 09.09.21 | - | Hitra, Norway | *Cancer pagurus* | 506 ± 127 | Width: 15.7 ± 0.8  Length: 10.0 ± 0.5 |
| **Brown crab, claws and brown meat,** boiled, 24 individuals  (= 8 composite samples) | Brown crab, claw meat | Delivered by fishermen | 2021 | Sampled: 08.11.21 | - | Austevoll, Norway | *Cancer pagurus* | - | - |
| **Crab claws**, pre-boiled, frozen, x6 claws (= 1 sample) | Brown crab, claw meat | Fish market | 2020 | Purchased: 20.10.20 | - | Hitra, Norway | *Cancer pagurus* | 931 | - |
| **Crab claws**, pre-boiled, frozen, x6 claws (= 1 sample) | Brown crab, claw meat | Fish market | 2021 | Purchased: 02.09.21 | - | Hitra, Norway | *Cancer pagurus* | 883 | - |
| **Crab claws**, pre-boiled, frozen, x6 claws (= 1 sample) | Brown crab, claw meat | Super-market | 2020 | Purchased: 21.10.20 | - | Hitra, Norway | *Cancer pagurus* | 529 | - |
| **Crab claws**, pre-boiled, fresh, “Lofoten”, x10 claws (= 1 sample) | Brown crab, claw meat | Super-market | 2020 | Produced: 13.10.20 | 23.10.20 | Hitra, Norway | *Cancer pagurus* | 460 | - |
| **Stuffed brown crab shell**, frozen, pre-boiled, “Kongshaugkrabbe” | Stuffed brown crab shells | Super-market | 2020 | Produced: 29.06.20 | 29.06.21 | Smøla, Norway | *Cancer pagurus* | 166 | Width: 12.8  Length: 8.5 |
| **Stuffed crab shell**, frozen, pre-boiled, “Kongshaugkrabbe” | Stuffed brown crab shells | Super-market | 2020 | Produced: 23.10.19 | 23.10.20 | Smøla, Norway | *Cancer pagurus* | 212 | Width: 14.9  Length: 9.2 |
| **Stuffed crab shell**, frozen, pre-boiled, “Kongshaugkrabbe” | Stuffed brown crab shells | Super-market | 2020 | Produced: 13.08.20 | 13.08.21 | Smøla, Norway | *Cancer pagurus* | 202 | Width: 15.4  Length: 9.6 |
| **Stuffed crab shell**, frozen, pre-boiled, “Kongshaugkrabbe” | Stuffed brown crab shells | Super-market | 2021 | Produced: 03.12.20 | 03.12.21 | Smøla, Norway | *Cancer pagurus* | 185 | Width: 14.0  Length: 8.5 |
| **Stuffed crab shell**, fresh, pre-boiled, “Åkra Sjømat” | Stuffed brown crab shells | Super-market | 2021 | Produced: 22.06.21 | 27.06.21 | FAO 27 | *Cancer pagurus* | 180 | Width: 14.4  Length: 9.1 |
| **Stuffed crab shell**, fresh, pre-boiled, “Åkra Sjømat” | Stuffed brown crab shells | Super-market | 2021 | Produced: 23.06.21 | 28.06.21 | FAO 27 | *Cancer pagurus* | 177 | Width: 14.4  Length: 8.8 |
| **Stuffed crab shell**, fresh, pre-boiled, “Åkra Sjømat” | Stuffed brown crab shells | Super-market | 2021 | Produced. 29.06.21 | 04.07.21 | FAO 27 | *Cancer pagurus* | 193 | Width: 15.0  Length: 9.3 |
| **Stuffed crab shell**, fresh, pre-boiled, “Åkra Sjømat” | Stuffed brown crab shells | Super-market | 2020 | Produced: 21.10.20 | 26.10.20 | FAO 27 | *Cancer pagurus* | 188 | Width: 14.2  Length: 9.0 |
| **Snow crabs,** claws and hepatopancreas, raw, 25 individuals (5 composite samples of each tissue, consisting of 5 individuals per composite sample) | Snow crab, leg meat  Snow crabs, hepatopancreas | Survey | 2021 | Sampled:  June 21 | - | The Barents Sea (75.8177N, 37.7145E) | *Chionoecetes opilio* | 419.0 ± 37.2 | Length: 82.9 ± 4.3 |
| **LOBSTERS AND CRAYFISH** |  |  |  |  |  |  |  |  |  |
| **Crayfish,** tail, boiled, frozen, “Øfas”, composite samples consisting of 20 individuals per sample | Crayfish, tails, boiled | Super-market | 2021 | 72212 | 09.22 | The river Guadalquivir, Spain | *Procambarus clarkii* | 34.0 ± 7.5 (mean weight per individual) | 4.3 ± 0.3 |
| **Crayfish,** tail, boiled, frozen, “Øfas”, composite samples consisting of 20 individuals per sample | Crayfish, tails, boiled | Super-market | 2021 | 75617 | 05.23 | The river Guadalquivir, Spain | *Procambarus clarkii* | 31.1 ± 6.1 (mean weight per individual) | 4.0 ± 0.2 |
| **Lobsters,** pre-boiled, frozen, “Star of the Sea”, 3 individuals, individual samples only | American lobster, white meat, boiled  American lobster, hepatopancreas, boiled | Super-market | 2021 | Produced: 08.09.20 | 08.09.23 | FAO 21 | *Homarus americanus* | 330 ± 20 | 8.1 ± 0.2 |
| **Lobster,** pre-boiled, frozen, “American lobster”, 3 individuals, individual samples only | American lobster, white meat, boiled  American lobster, hepatopancreas, boiled | Super-market | 2021 | 2391 (Produced: 15.10.20) | 15.10.23 | FAO 21 | *Homarus americanus* | 323 ± 21 | 8.3 ± 0.2 |
| **Lobster,** pre-boiled, frozen, 3 individuals, individual samples only | American lobster, white meat, boiled  American lobster, hepatopancreas, boiled | Super-market | 2021 | - | Purchased: 15.09.21 | FAO 21 | *Homarus americanus* | 273 ± 37 | 7.9 ± 0.4 |
| **Lobster,** boiled, frozen, 28 individuals (= 4 composite samples ) | Norway lobster, white meat, boiled | Delivered by fishermen | 2021 | Sampled: 29.11.21 | - | Austevoll, Norway | *Nephrops norvegicus* | - | - |
| **Lobster,** raw, 25 individuals; composite samples (consisting of 5 individuals) | Norway lobster, white meat, raw  Norway lobster, hepatopancreas, raw | Survey | 2021 | Sampled: 11.10.21 | - | Årdalsfjorden, Sognefjorden, Norway | *Nephrops norvegicus* | 104.6 ± 49.3 | 5.8 ± 0.7 |
| **Lobster,** raw, 25 individuals; composite samples (consisting of 5 individuals) | Norway lobster, white meat, raw  Norway lobster, hepatopancreas, raw | Survey | 2021 | Sampled: 11.08.21 | - | Eidsfjorden, Norway | *Nephrops norvegicus* | - | - |
| **Lobsters,** raw**,** 20 individuals; composite samples (consisting of 4 individuals) | European lobster, white meat, raw  European lobster, hepatopancreas, raw | Delivered by fishermen | 2021 | Sampled: 01.11.21 | - | Austevoll, Norway | *Homarus gammarus* | 625.2 ± 151 | 9.77 ± 6.0 |
| **Lobsters,** boiled**,** 20 individuals: composite samples (consisting of 4 individuals) | European lobster, white meat, boiled  European lobster, hepatopancreas, boiled | Delivered by fishermen | 2021 | Sampled: 01.11.21 | - | Austevoll, Norway | *Homarus gammarus* | 697.0 ± 258 | 9.93 ± 9.1 |
| **Lobsters,** raw**,** 20 individuals; composite samples (consisting of 4 individuals) | European lobster, white meat, raw  European lobster, hepatopancreas, raw | Delivered by fishermen | 2021 | Sampled: 07.11.21 | - | Austevoll, Norway | *Homarus gammarus* | 622.4 ± 298 | 9.63 ± 1.0 |
| **Lobsters,** boiled**,** 20 individuals: composite samples (consisting of 4 individuals) | European lobster, white meat, boiled  European lobster, hepatopancreas, boiled | Delivered by fishermen | 2021 | Sampled: 07.11.21 | - | Austevoll, Norway | *Homarus gammarus* | 434.5 ± 50.4 | 9.20 ± 0.3 |
| **SHRIMPS** |  |  |  |  |  |  |  |  |  |
| **Shrimps**, pre-boiled, (hand-peeled during sample prep.), 85 individuals | Northern shrimps, peeled | Fish market | 2020 | Purchased: 20.10.20 | - | Sirevåg, Rogaland, Norway | *Pandalus borealis* | 552 | 4.7 ± 0.4 |
| **Shrimps**, pre-boiled, (hand-peeled during sample prep.), “Royal Greenland”, 64 individuals | Northern shrimps, peeled | Super-market | 2020 | Purchased: 20.10.20 | - | FAO 27 | *Pandalus borealis* | 750 | 5.6 ± 0.3 |
| **Shrimps**, pre-boiled, (hand-peeled during sample prep.), “Royal Greenland”, 85 individuals | Northern shrimps, peeled | Super-market | 2021 | Purchased: 31.08.21 | December 2022 | FAO 27 | *Pandalus borealis* | 958 | 5.5 ± 0.3 |
| **Shrimps**, pre-boiled, (hand-peeled during sample prep.), “Polar Seafood Greenland”, 50 individuals | Northern shrimps, peeled | Super-market | 2021 | 01.2020 | 28.01.22 | FAO 21/27 | *Pandalus borealis* | 533 | 5.3 ± 0.3 |
| **Shrimps**, pre-boiled, (hand-peeled during sample prep.), “Greenland Prawns, Iceberg Seafood AS”, 85 individuals | Northern shrimps, peeled | Super-market | 2021 | 06.2020 | 17.05.22 | FAO 27 | *Pandalus borealis* | 974 | 5.6 ± 0.3 |
| Hand-peeled **shrimps in brine**, “Fiskemannen” | Northern shrimps, in brine | Super-market | 2020 | - | 29.11.20 | FAO 21 | *Pandalus borealis* | 311 | - |
| Hand-peeled **shrimps in brine**, “Fiskemannen” | Northern shrimps, in brine | Super-market | 2020 | - | 16.11.20 | FAO 21 | *Pandalus borealis* | 306 | - |
| Hand-peeled **shrimps in brine**, “Fiskemannen” | Northern shrimps, in brine | Super-market | 2020 | - | 09.12.20 | FAO 21 | *Pandalus borealis* | 302 | - |
| Hand-peeled **shrimps in brine**, “Fiskemannen” | Northern shrimps, in brine | Super-market | 2021 | - | 30.09.21 | FAO 21 | *Pandalus borealis* | 205 | - |
| Hand-peeled **shrimps in brine**, “Fiskemannen” | Northern shrimps, in brine | Super-market | 2021 | - | 12.10.21 | FAO 21 | *Pandalus borealis* | 199 | - |
| Hand-peeled **shrimps in brine**, “Fiskemannen” | Northern shrimps, in brine | Super-market | 2021 | - | 18.10.21 | FAO 21 | *Pandalus borealis* | 202 | - |
| **Shrimps in brine**, “Fiskemannen” | Northern shrimps, in brine | Super-market | 2020 | 11:35, L4 | 16.11.20 | FAO 21 | *Pandalus borealis* | 202 | - |
| **Shrimps in brine**, “Fiskemannen” | Northern shrimps, in brine | Super-market | 2020 | 11:38, L4 | 10.11.20 | FAO 21 | *Pandalus borealis* | 209 | - |
| **Shrimps in brine**, “Fiskemannen” | Northern shrimps, in brine | Super-market | 2020 | 12:28, L4 | 30.11.20 | FAO 21 | *Pandalus borealis* | 185 | - |
| **Shrimps in brine**, “Fiskemannen” | Northern shrimps, in brine | Super-market | 2021 | 15:33, L4 | 21.09.21 | FAO 21 | *Pandalus borealis* | 197 | - |
| **Shrimps in brine**, “Fiskemannen” | Northern shrimps, in brine | Super-market | 2021 | 12:40, L4 | 28.09.21 | FAO 21 | *Pandalus borealis* | 193 | - |
| **Shrimps in brine**, “Fiskemannen” | Northern shrimps, in brine | Super-market | 2021 | 12:03, L4 | 03.10.21 | FAO 21 | *Pandalus borealis* | 191 | - |
| Freshly peeled **shrimps** from Sørlandet, “Fiskemannen” | Northern shrimps, peeled | Super-market | 2020 | 2600847 | 16.11.20 | FAO 27 | *Pandalus borealis* | 97 | - |
| Freshly peeled **shrimps** from Sørlandet, “Fiskemannen” | Northern shrimps, peeled | Super-market | 2020 | 2681056 | 23.11.20 | FAO 27 | *Pandalus borealis* | 97 | - |
| Freshly peeled **shrimps** from Sørlandet, “Fiskemannen” | Northern shrimps, peeled | Super-market | 2020 | 2731507 | 28.11.20 | FAO 27 | *Pandalus borealis* | 97 | - |
| Freshly peeled **shrimps** from Sørlandet, “Fiskemannen” | Northern shrimps, peeled | Super-market | 2021 | 21511367 | 09.10.21 | FAO 27 | *Pandalus borealis* | 98 | - |
| Freshly peeled **shrimps** from Sørlandet, “Fiskemannen” | Northern shrimps, peeled | Super-market | 2021 | 2310909 | 18.10.21 | FAO 27 | *Pandalus borealis* | 101 | - |
| Freshly peeled **shrimps** from Sørlandet, “Fiskemannen” | Northern shrimps, peeled | Super-market | 2021 | 2181124 | 25.10.21 | FAO 27 | *Pandalus borealis* | 97 | - |
| Peeled **shrimps**, frozen, “Fiskemannen” | Northern shrimps, peeled | Super-market | 2020 | Produced: 10.01.20 | 10.07.21 | FAO 27 | *Pandalus borealis* | 142 | - |
| Peeled **shrimps**, frozen, “Fiskemannen” | Northern shrimps, peeled | Super-market | 2020 | Produced: 11.01.20 | 11.07.21 | FAO 27 | *Pandalus borealis* | 141 | - |
| Peeled **shrimps**, frozen, “Fiskemannen” | Northern shrimps, peeled | Super-market | 2021 | Produced: 01.12.20 | 01.06.22 | FAO 27 | *Pandalus borealis* | 162 | - |
| Peeled **shrimps**, frozen, “Fiskemannen” | Northern shrimps, peeled | Super-market | 2021 | Produced: 08.04.21 | 08.10.22 | FAO 27 | *Pandalus borealis* | 166 | - |
| **Shrimps in brine**, “First Price” | Northern shrimps, in brine | Super-market | 2020 | 07:06, L2 | 02.12.20 | FAO 67/21/27 | *Pandalus borealis/jordani* | 380 | - |
| **Shrimps in brine**, “First Price” | Northern shrimps, in brine | Super-market | 2020 | 06:33, L2 | 11.11.20 | FAO 67/21/27 | *Pandalus borealis/jordani* | 382 | - |
| **Shrimps in brine**, “First Price” | Northern shrimps, in brine | Super-market | 2020 | 06:32, L2 | 18.11.20 | FAO 67/21/27 | *Pandalus borealis/jordani* | 373 | - |
| **Shrimps in brine**, “First Price” | Northern shrimps, in brine | Super-market | 2021 | 09:08, L2 | 15.09.21 | FAO 67/21/27 | *Pandalus borealis/jordani* | 375 | - |
| **Shrimps in brine**, “First Price” | Northern shrimps, in brine | Super-market | 2021 | 11:21, L2 | 21.09.21 | FAO 67/21/27 | *Pandalus borealis/jordani* | 396 | - |
| **Shrimps in brine**, “First Price” | Northern shrimps, in brine | Super-market | 2021 | 06:07, L2 | 28.09.21 | FAO 67/21/27 | *Pandalus borealis/jordani* | 366 | - |
| **Shrimps**, whole, frozen, in bag (hand-peeled during sample prep.), “First Price”, 85 individuals (=1 sample) | Northern shrimps, peeled | Super-market | 2020 | P7994 | 22.06.21 | Greenland | *Pandalus borealis* | 756 | 5.1 ± 0.4 |
| **Shrimps**, whole, frozen, in bag (hand-peeled during sample prep.), “First Price”, 85 individuals (=1 sample) | Northern shrimps, peeled | Super-market | 2020 | P7989 | 13.05.21 | Greenland | *Pandalus borealis* | 628 | 4.8 ± 0.3 |
| **Shrimps**, whole, frozen, in bag (hand-peeled during sample prep.), “First Price”, 85 individuals (=1 sample) | Northern shrimps, peeled | Super-market | 2021 | 2001122 | 30.12.22 | Greenland | *Pandalus borealis* | 711 | 5.0 ± 0.4 |
| **Shrimps**, whole, frozen, in bag (hand-peeled during sample prep.), “First Price”, 85 individuals (=1 sample) | Northern shrimps, peeled | Super-market | 2021 | 2011127 | 01.01.23 | Greenland | *Pandalus borealis* | 729 | 5.0 ± 0.3 |
| **Shrimps** with shell from Greenland, frozen, (hand-peeled during sample prep.), “Xtra”, 85 individuals (=1 sample) | Northern shrimps, peeled | Super-market | 2020 | Produced: 15.06.20 | 12.03.21 | FAO 21 | *Pandalus borealis* | 711 | 5.1 ± 0.3 |
| **Shrimps** with shell from Greenland, frozen, (hand-peeled during sample prep.), “Xtra”, 85 individuals (=1 sample) | Northern shrimps, peeled | Super-market | 2020 | Produced: 27.07.20 | 23.04.21 | FAO 21 | *Pandalus borealis* | 701 | 5.1 ± 0.3 |
| **Shrimps** with shell from Greenland, frozen, (hand-peeled during sample prep.), “Xtra”, 85 individuals (=1 sample) | Northern shrimps, peeled | Super-market | 2021 | Produced: 20.05.21 | 14.02.22 | FAO 21 | *Pandalus borealis* | 717 | 4.8 ± 0.4 |
| **Shrimps** with shell from Greenland, frozen, (hand-peeled during sample prep.), “Xtra”, 85 individuals (=1 sample) | Northern shrimps, peeled | Super-market | 2021 | Produced: 08.04.21 | 03.01.22 | FAO 21 | *Pandalus borealis* | 646 | 4.5 ± 0.3 |
| **Shrimps in brine**, “Xtra” | Northern shrimps, in brine | Super-market | 2020 | Produced: A15 09.20 | 04.11.21 | FAO 67 | *Pandalus jordani* | 397 | - |
| **Shrimps in brine**, “Xtra” | Northern shrimps, in brine | Super-market | 2020 | Produced: A21 10.20 | 10.12.20 | FAO 67 | *Pandalus jordani* | 363 | - |
| **Shrimps in brine**, “Xtra” | Northern shrimps, in brine | Super-market | 2020 | Produced: A22 09.20 | 11.11.20 | FAO 67 | *Pandalus jordani* | 418 | - |
| **Shrimps in brine**, “Xtra” | Northern shrimps, in brine | Super-market | 2021 | DKEF 4661, 0937 | 04.10.21 | FAO 67 | *Pandalus jordani* | 402 | - |
| **Shrimps in brine**, “Xtra” | Northern shrimps, in brine | Super-market | 2021 | DKEF 4661, 1118 | 26.10.21 | FAO 67 | *Pandalus jordani* | 400 | - |
| **Shrimps in brine**, “Xtra” | Northern shrimps, in brine | Super-market | 2021 | DKEF 4331, 1143 | 03.12.21 | FAO 67 | *Pandalus jordani* | 434 | - |
| Norwegian **shrimps in brine**, “Sirevaag” | Northern shrimps, in brine | Super-market | 2020 | 8226 | 18.11.20 | FAO 27 | *Pandalus borealis* | 173 | - |
| Norwegian **shrimps in brine**, “Sirevaag” | Northern shrimps, in brine | Super-market | 2020 | 8238 | 02.12.20 | FAO 27 | *Pandalus borealis* | 176 | - |
| Norwegian **shrimps in brine,** “Sirevaag” | Northern shrimps, in brine | Super-market | 2020 | 8253 | 21.12.20 | FAO 27 | *Pandalus borealis* | 172 | - |
| Norwegian **shrimps in brine,** “Sirevaag” | Northern shrimps, in brine | Super-market | 2021 | 8445 | 11.10.21 | FAO 27 | *Pandalus borealis* | 176 | - |
| Norwegian **shrimps in brine**, “Sirevaag” | Northern shrimps, in brine | Super-market | 2021 | 8434 | 28.09.21 | FAO 27 | *Pandalus borealis* | 178 | - |
| Norwegian **shrimps in brine,** “Sirevaag” | Northern shrimps, in brine | Super-market | 2021 | 8452 | 18.10.21 | FAO 27 | *Pandalus borealis* | 186 | - |
| Hand peeled **shrimps in brine**, “Engelsviken” | Northern shrimps, in brine | Super-market | 2020 | 3-33, 2020 230 | 15.11.20 | Greenland | *Pandalus borealis* | 314 | - |
| Hand peeled **shrimps in brine,** “Engelsviken” | Northern shrimps, in brine | Super-market | 2020 | 2-34, 2020 237 | 22.11.29 | Greenland | *Pandalus borealis* | 317 | - |
| Hand peeled **shrimps in brine,** “Engelsviken” | Northern shrimps, in brine | Super-market | 2020 | 60-049, 2020 241 | 03.12.20 | Greenland | *Pandalus borealis* | 316 | - |
| Hand peeled **shrimps in brine**, “Engelsviken” | Northern shrimps, in brine | Super-market | 2021 | 60-032, 2021-195 | 22.10.21 | Greenland | *Pandalus borealis* | 295 | - |
| Hand peeled **shrimps in brine**, “Engelsviken” | Northern shrimps, in brine | Super-market | 2021 | 60-043, 2021 247 | 08.12.21 | Greenland | *Pandalus borealis* | 290 | - |
| Hand peeled **shrimps in brine**, “Engelsviken” | Northern shrimps, in brine | Super-market | 2021 | 60-053, 2021 266 | 01.01.22 | Greenland | *Pandalus borealis* | 299 | - |
| **Shrimps in brine**, “Prima” | Northern shrimps, in brine | Super-market | 2020 | - | 18.11.20 | FAO 67/21 | *Pandalus borealis/jordani* | 406 | - |
| **Shrimps in brine**, “Prima” | Northern shrimps, in brine | Super-market | 2020 | - | 25.11.20 | FAO 67/21 | *Pandalus borealis/jordani* | 403 | - |
| **Shrimps in brine**, “Prima” | Northern shrimps, in brine | Super-market | 2020 | - | 02.12.20 | FAO 67/21 | *Pandalus borealis/jordani* | 396 | - |
| **Shrimps in brine**, “Prima” | Northern shrimps, in brine | Super-market | 2021 | - | 29.09.21 | FAO 67/21 | *Pandalus borealis/jordani* | 436 | - |
| **Shrimps in brine**, “Prima” | Northern shrimps, in brine | Super-market | 2021 | - | 11.20.21 | FAO 67/21 | *Pandalus borealis/jordani* | 396 | - |
| **Shrimps in brine**, “Prima” | Northern shrimps, in brine | Super-market | 2021 | - | 22.09.21 | FAO 67/21 | *Pandalus borealis/jordani* | 386 | - |
| Hand-peeled **shrimps in brine**, “Fiskeriet” | Northern shrimps, in brine | Super-market | 2020 | - | 05.11.20 | FAO 21 | *Pandalus borealis* | 202 | - |
| Hand-peeled **shrimps in brine,** “Fiskeriet” | Northern shrimps, in brine | Super-market | 2020 | - | 02.12.20 | FAO 21 | *Pandalus borealis* | 191 | - |
| Hand-peeled **shrimps in brine,** “Fiskeriet” | Northern shrimps, in brine | Super-market | 2020 | - | 10.11.20 | FAO 21 | *Pandalus borealis* | 199 | - |
| Hand-peeled s**hrimps in brine,** “Fiskeriet” | Northern shrimps, in brine | Super-market | 2021 | - | 29.09.21 | FAO 21 | *Pandalus borealis* | 206 | - |
| Hand-peeled **shrimps in brine**, “Fiskeriet” | Northern shrimps, in brine | Super-market | 2021 | - | 18.12.21 | FAO 21 | *Pandalus borealis* | 205 | - |
| Hand-peeled **shrimps in brine,** “Fiskeriet” | Northern shrimps, in brine | Super-market | 2021 | - | 24.12.21 | FAO 21 | *Pandalus borealis* | 212 | - |
| **Shrimps** 90+, frozen, (hand-peeled during sample prep.), “Prima”, 85 individuals (=1 sample) | Northern shrimps, peeled | Super-market | 2020 | 326201 (Produced: 17.01.20) | 17.01.22 | FAO 21 | *Pandalus borealis* | 562 | 4.8 ± 0.3 |
| **Shrimps** 90+, frozen, (hand-peeled during sample prep.), “Prima”, 85 individuals (=1 sample) | Northern shrimps, peeled | Super-market | 2021 | 418211 (Produced: 17.03.21) | 17.03.23 | FAO 21 | *Pandalus borealis* | 666 | 4.4 ± 0.3 |
| **Shrimps** 90+, frozen, (hand-peeled during sample prep.), “Prima”, 85 individuals (=1 sample) | Northern shrimps, peeled | Super-market | 2021 | Produced: 04.03.21 | 04.03.23 | FAO 21 | *Pandalus borealis* | 652 | 4.6 ± 0.3 |
| **Shrimps** 90+, frozen, (hand-peeled during sample prep.), “Prima”, 85 individuals (=1 sample) | Northern shrimps, peeled | Super-market | 2021 | 2634211 (Produced: 19.06.21) | 19.06.23 | FAO 21 | *Pandalus borealis* | 650 | 4.7 ± 0.3 |
| **Shrimps in brine**, “Fiskeriet” | Northern shrimps, in brine | Super-market | 2020 | - | 02.12.20 | FAO 21/27 | *Pandalus borealis* | 201 | - |
| **Shrimps in brine**, “Fiskeriet” | Northern shrimps, in brine | Super-market | 2020 | - | 24.11.20 | FAO 21/27 | *Pandalus borealis* | 221 | - |
| **Shrimps in brine**, “Fiskeriet” | Northern shrimps, in brine | Super-market | 2020 | - | 17.11.20 | FAO 21/27 | *Pandalus borealis* | 234 | - |
| Fresh Norwegian **shrimps in brine**, “Reimes” | Northern shrimps, in brine | Super-market | 2020 | 8214 | 04.11.20 | FAO 27 | *Pandalus borealis* | 302 | - |
| Fresh Norwegian **shrimps in brine**, “Reimes” | Northern shrimps, in brine | Super-market | 2020 | 8233 | 25.11.20 | FAO 27 | *Pandalus borealis* | 304 | - |
| Fresh Norwegian **shrimps in brine**, “Reimes” | Northern shrimps, in brine | Super-market | 2020 | 8243 | 16.12.20 | FAO 27 | *Pandalus borealis* | 294 | - |
| Fresh Norwegian **shrimps in brine**, “Reimes” | Northern shrimps, in brine | Super-market | 2021 | 8431 | 21.09.21 | FAO 27 | *Pandalus borealis* | 304 | - |
| Fresh Norwegian **shrimps in brine**, “Reimes” | Northern shrimps, in brine | Super-market | 2021 | 8433 | 23.09.21 | FAO 27 | *Pandalus borealis* | 303 | - |
| Fresh Norwegian **shrimps in brine**, “Reimes” | Northern shrimps, in brine | Super-market | 2021 | 8449 | 13.10.21 | FAO 27 | *Pandalus borealis* | 304 | - |
| **Shrimps**, boiled, frozen, whole/unpeeled, 25 individuals (=1 sample) | Northern shrimps, unpeeled | Survey | 2021 | - | Sampled: 14.01.21 | The North Sea (58.9688N, 5.1729E) | *Pandalus borealis* | 6.1 (mean weight per shrimp) | 10.8^d^ |
| **Shrimps**, boiled, hand-peeled, frozen, 25 individuals per sample (x3 composite samples) | Northern shrimps, peeled | Survey | 2021 | - | Sampled: 14.01.21 | The North Sea (58.9688N, 5.1729E) | *Pandalus borealis* | 5.9 (mean weight per shrimp) | 10.7^d^ |
| **Shrimps**, boiled, frozen, whole/unpeeled, 25 individuals (=1 sample) | Northern shrimps, unpeeled | Survey | 2021 | - | Sampled: 24.01.21 | The North Sea (58.2045N, 8.5680E) | *Pandalus borealis* | 5.4 (mean weight per shrimp) | 11.1^d^ |
| **Shrimps**, boiled, hand-peeled, frozen 25 individuals per sample (x3 composite samples) | Northern shrimps, peeled | Survey | 2021 | - | Sampled: 24.01.21 | The North Sea (58.2045N, 8.5680E) | *Pandalus borealis* | 4.4 (mean weight per shrimp) | 10.1^d^ |
| **Shrimps**, boiled, frozen, whole/unpeeled, 25 individuals (=1 sample) | Northern shrimps, unpeeled | Survey | 2021 | - | Sampled: 24.01.21 | The Barents Sea (7455.7252N, 01654.5513E) | *Pandalus borealis* | 3.8 (mean weight per shrimp) | 10.1^d^ |
| **Shrimps**, boiled, hand-peeled, frozen 25 individuals per sample (x3 composite samples) | Northern shrimps, peeled | Survey | 2021 | - | Sampled: 24.01.21 | The Barents Sea  (7455.7252N, 01654.5513E) | *Pandalus borealis* | 3.5 (mean weight per shrimp) | 10.4^d^ |
| **Shrimps**, boiled, frozen, whole/unpeeled, 25 individuals (=1 sample) | Northern shrimps, unpeeled | Survey | 2021 | - | Sampled: 24.01.21 | The Barents Sea (7710.8388N, 01257.6703E) | *Pandalus borealis* | - | - |
| **Shrimps**, boiled, hand-peeled, frozen 25 individuals per sample (x3 composite samples) | Northern shrimps, peeled | Survey | 2021 | - | Sampled: 24.01.21 | The Barents Sea  (7710.8388N, 01257.6703E) | *Pandalus borealis* | - | - |
| **Shrimps**, boiled, frozen, whole/unpeeled, 25 individuals (=1 sample) | Northern shrimps, unpeeled | Survey | 2021 | - | Sampled: 06.02.21 | The Barents Sea/The Greenland Sea (7710.8388N, 01257.6703E) | *Pandalus borealis* | 5.3 (mean weight per shrimp) | 10.1^d^ |
| **Shrimps**, boiled, hand-peeled, frozen 25 individuals per sample (x3 composite samples) | Northern shrimps, peeled | Survey | 2021 | - | Sampled: 06.02.21 | The Barents Sea/The Greenland Sea (7710.8388N, 01257.6703E) | *Pandalus borealis* | 6.2 (mean weight per shrimp) | 10.5^d^ |
| **Shrimps**, boiled, frozen, whole/unpeeled, 25 individuals (=1 sample) | Northern shrimps, unpeeled | Survey | 2021 | - | Sampled: 15.11.21 | Foldafjorden (64.56180N, 11.59160E) | *Pandalus borealis* | 7.5 (mean weight per shrimp) | 2.4^e^ |
| **Shrimps**, boiled, hand-peeled, frozen 25 individuals per sample (x3 composite samples) | Northern shrimps, peeled | Survey | 2021 | - | Sampled: 15.11.21 | Foldafjorden (64.56180N, 11.59160E) | *Pandalus borealis* | 8.6 (mean weight per shrimp) | 2.4^e^ |
| **Shrimps**, boiled, frozen, whole/unpeeled, 25 individuals (=1 sample) | Northern shrimps, peeled | Survey | 2021 | - | Sampled: 18.11.21 | Foldafjorden (64.93633N, 11.98600E) | *Pandalus borealis* | 6.7 (mean weight per shrimp) | 2.2^e^ |
| **Shrimps**, boiled, hand-peeled, frozen 25 individuals per sample (x3 composite samples) | Northern shrimps, peeled | Survey | 2021 | - | Sampled: 18.11.21 | Foldafjorden (64.93633N, 11.98600E) | *Pandalus borealis* | 5.8 (mean weight per shrimp) | 2.1^e^ |

^a^ Describing the categorisation of each shellfish product, as used in the article. The various shellfish categories and their corresponding processing methods are described in **Table 1** in the article.
^b^ FAO ‘number’ corresponds to the FAO Major Fishing Areas (<https://www.fao.org/fishery/en/area/search>)
^c^ Weights and lengths are provided as mean weight and mean length (± standard deviation) for shellfish products that were measured individually. For shellfish in brine and pre-peeled shellfish, the weight and length are provided for one batch of each shellfish product (regardless of how many portions one batch contains). Physical parameters were not available for all sampled shellfish products (marked with a hyphen, “-“).
^d^ Measured as the length of the whole shrimp: the length from the corner of the eye to the posterior end of the tail.
^e^ Measured as the length of the cephalothorax: the length from the corner of the eye to the posterior end of the carapace where the [tail](https://www.sciencedirect.com/topics/agricultural-and-biological-sciences/tail) begins.
**Abbreviations**: FAO: Food and Agriculture Organization of the United Nations; NFSA: Norwegian Food Safety Authority.
