# Supplementary material for: Quantification of vitamin K (phylloquinone and menaquinones 4–10) in various shellfish
Source: Br J Nutr. 2025 Feb 13;133(4):469–80. doi: 10.1017/S0007114525000261 (PMC12011545; doi:10.1017/S0007114525000261)
Supplement: Moxness Reksten et al. supplementary material 2 — Moxness Reksten et al. supplementary material [file S0007114525000261sup002.docx]

**Table 2**: Overview of the limits of quantification (LOQ, µg/100 g) for each vitamer, assuming 1 g of sample material and 0.5 mL of final solvent.

| **Aliquot^a^** | **Injection volume^b^** | **MK-4** | **MK-5** | **K1** | **ß, ϒ-2H-K_1_** | **MK-6** | **MK-7** | **MK-8** | **MK-9** | **MK-10** |
| --- | --- | --- | --- | --- | --- | --- | --- | --- | --- | --- |
| **1/3** | **2.5** | 0.12 | 0.18 | 0.18 | 0.10 | 0.30 | 0.12 | 0.18 | 0.24 | 0.42 |
| **1/3** | **5** | 0.060 | 0.090 | 0.090 | 0.090 | 0.15 | 0.060 | 0.090 | 0.120 | 0.21 |
| **1/3** | **10** | 0.030 | 0.045 | 0.045 | 0.045 | 0.075 | 0.030 | 0.045 | 0.060 | 0.11 |
| **2/3** | **2.5** | 0.060 | 0.090 | 0.090 | 0.090 | 0.15 | 0.060 | 0.090 | 0.12 | 0.21 |
| **2/3** | **5** | 0.030 | 0.045 | 0.045 | 0.045 | 0.075 | 0.030 | 0.045 | 0.060 | 0.11 |
| **2/3** | **10** | 0.015 | 0.023 | 0.023 | 0.023 | 0.038 | 0.015 | 0.023 | 0.030 | 0.053 |

^a^ The aliquot refers to the amount of the hexane phase that is transferred, where either 1/3 or 2/3 of the hexane phase is transferred depending on the expected vitamin K content in the sample.
^b^ The injection volume refers to the quantity of the sample that is injected (µl) by the autosampler into the HPLC-FLD system. Samples are injected with volumes ranging from 1 to 20 µl, depending on the expected concentration of the highest vitamer in the sample. In this study, samples were only injected with volumes ranging from 2.5 to 10 µl.  **Abbreviations**: ß, ϒ-2H-K_1_**:** beta, gamma-dihydrophylloquinone; HPLC-FLD: high pressure liquid chromatography with fluorescence detector; LOQ: limit of quantification; MK: menaquinone.
