# Supplementary material for: Quantification of vitamin K (phylloquinone and menaquinones 4–10) in various shellfish
Source: Br J Nutr. 2025 Feb 13;133(4):469–80. doi: 10.1017/S0007114525000261 (PMC12011545; doi:10.1017/S0007114525000261)
Supplement: Moxness Reksten et al. supplementary material 3 — Moxness Reksten et al. supplementary material [file S0007114525000261sup003.docx]

**Table 3**: The number of samples with values below the limit of quantification (LOQ) for each shellfish product. The percentage of samples with values <LOQ is presented in the parentheses, whereas a hyphen (-) indicates no values below the LOQ.

| **Shellfish product** | **Total** | **Phylloquinone** | **ß, ϒ-2H-K_1_** | **MK-4** | **MK-5** | **MK-6** | **MK-7** | **MK-8** | **MK-9** | **MK-10** |
| --- | --- | --- | --- | --- | --- | --- | --- | --- | --- | --- |
|  | **n** | **(K_1_)** | **(K_1_)** | **(K_2_)** | **(K_2_)** | **(K_2_)** | **(K_2_)** | **(K_2_)** | **(K_2_)** | **(K_2_)** |
| **BLUE MUSSELS** |  |  |  |  |  |  |  |  |  |  |
| Blue mussels, raw | 49 | 3  (6%) | 49  (100%) | 14  (29%) | 42  (86%) | 19  (39%) | 19  (39%) | 28  (57%) | 47  (96%) | 49  (100%) |
| Blue mussels, steamed | 8 | - | 8  (100%) | - | 2  (25%) | - | - | 2  (25%) | 7  (88%) | 8  (100%) |
| Blue mussels, in brine | 4 | - | 4  (100%) | - | - | - | - | - | 3  (75%) | 3  (75%) |
| Blue mussels, pre-packaged | 4 | - | 4  (100%) | - | - | - | - | 1  (25%) | 3  (75%) | 3  (75%) |
| **SCALLOPS** |  |  |  |  |  |  |  |  |  |  |
| Deep sea scallop, raw | 7 | - | 7  (100%) | - | 2  (29%) | 6  (86%) | 7  (100%) | 7  (100%) | 7  (100%) | 7  (100%) |
| Great scallop, raw | 10 | 10  (100%) | 10  (100%) | 1  (10%) | 10  (100%) | 10  (100%) | 9  (90%) | 7  (70%) | 10  (100%) | 10  (100%) |
| **SHRIMPS** |  |  |  |  |  |  |  |  |  |  |
| Northern shrimps, peeled | 48 | 8  (17%) | 48  (100%) | 1  (2%) | 42  (88%) | 17  (35%) | 9  (19%) | 29  (60%) | 48  (100%) | 48  (100%) |
| Northern shrimps, unpeeled | 7 | - | 7  (100%) | - | 1  (14%) | - | - | - | 2  (29%) | 5  (71%) |
| Northern shrimps, in brine | 57 | 3  (5%) | 57  (100%) | 5  (9%) | 57  (100%) | 57  (100%) | 41  (72%) | 54  (95%) | 57  (100%) | 57  (100%) |
| **CRABS** |  |  |  |  |  |  |  |  |  |  |
| Brown crab, claw meat | 18 | 1  (5%) | 18  (100%) | 17  (94%) | 18  (100%) | 11  (61%) | 9  (50%) | 16  (89%) | 18  (100%) | 18  (100%) |
| Brown crab, brown meat | 14 | - | 14  (100%) | - | - | - | - | - | - | 2  (14%) |
| Stuffed brown crab shells | 7 | - | 7  (100%) | 1  (14%) | - | - | - | - | 1  (14%) | 1  (14%) |
| Snow crab, leg meat | 5 | 2  (40%) | 5  (100%) | 2  (40%) | 5  (100%) | 5  (100%) | - | 5  (100%) | 5  (100%) | 5  (100%) |
| Snow crab, hepatopancreas | 5 | - | 5  (100%) | - | - | - | - | - | - | - |
| **LOBSTERS & CRAYFISH** |  |  |  |  |  |  |  |  |  |  |
| Crayfish, tails, boiled | 2 | - | 2  (100%) | 2  (100%) | 1  (50%) | 1  (50%) | - | - | 2  (100%) | 2  (100%) |
| Norway lobster, white meat, raw | 3 | 2  (67%) | 3  (100%) | 1  (33%) | 3  (100%) | 3  (100%) | 2  (67%) | 3  (100%) | 3  (100%) | 3  (100%) |
| Norway lobster, white meat, boiled | 4 | 2  (50%) | 4  (100%) | 4  (100%) | 4  (100%) | 4  (100%) | 1  (25%) | 3  (75%) | 4  (100%) | 4  (100%) |
| Norway lobster, hepatopancreas, raw | 5 | - | 5  (100%) | 4  (80%) | - | - | - | - | - | 5  (100%) |
| American lobster, white meat, boiled | 6 | 3  (50%) | 6  (100%) | 2  (33%) | 3  (50%) | 3  (50%) | 2  (33%) | 4  (67%) | 6  (100%) | 6  (100%) |
| American lobster, hepatopancreas, boiled | 3 | - | 3  (100%) | - | - | - | - | - | 3  (100%) | 3  (100%) |
| European lobster, white meat, raw | 20 | - | 20  (100%) | - | 20  (100%) | 8  (40%) | 2  (10%) | 10  (50%) | 13  (65%) | 20  (100%) |
| European lobster, hepatopancreas, raw | 10 | - | 10  (100%) | 2  (20%) | - | - | - | - | - | 10  (100%) |
| European lobster, white meat, boiled | 19 | 1  (5%) | 19  (100%) | 3  (16%) | 19  (100%) | 10  (53%) | - | 10  (53%) | 19  (100%) | 19  (100%) |
| European lobster, hepatopancreas, boiled | 8 | 1  (13%) | 8  (100%) | 2  (25%) | 3  (38%) | - | - | 1  (13%) | 5  (63%) | 8  (100%) |
| **Total** | 323 | 36  (11%) | 324  (100%) | 61  (19%) | 232  (72%) | 154  (48%) | 101  (31%) | 180  (56%) | 263  (81%) | 296  (91%) |
