# Supplementary material for: Quantification of vitamin K (phylloquinone and menaquinones 4–10) in various shellfish
Source: Br J Nutr. 2025 Feb 13;133(4):469–80. doi: 10.1017/S0007114525000261 (PMC12011545; doi:10.1017/S0007114525000261)
Supplement: Moxness Reksten et al. supplementary material 4 — Moxness Reksten et al. supplementary material [file S0007114525000261sup004.docx]

**Table 4**: Contents of phylloquinone, beta, gamma-dihydrophylloquinone (dihydro K_1_), and menaquinones (MK-4 to MK-10) in various shellfish products. The values are reported as means ± standards deviations (SD) on the first line and ranges (minimum-maximum) on the second line. All values are expressed in µg/100 g wet weight^a^

| **Shellfish product** | **Phylloquinone** | **ß, ϒ-2H-K_1_** | **MK-4** | **MK-5** | **MK-6** | **MK-7** | **MK-8** | **MK-9** | **MK-10** | **Total** |
| --- | --- | --- | --- | --- | --- | --- | --- | --- | --- | --- |
|  | **(K_1_)** | **(K_1_)^b^** | **(K_2_)** | **(K_2_)** | **(K_2_)** | **(K_2_)** | **(K_2_)** | **(K_2_)** | **(K_2_)** | **(K_1_ + K_2_)** |
| **BLUE MUSSELS** |  |  |  |  |  |  |  |  |  |  |
| Blue mussels, raw | 0.52 ± 0.30  0.090^c^-1.45 | 0.090 ± 0.00  0.090^c^-0.090 | 0.28 ± 0.29  0.060^c^-1.69 | 0.11 ± 0.053  0.090^c^-0.26 | 0.45 ± 0.37  0.15^c^-1.65 | 0.22 ± 0.25  0.060^c^-1.52 | 0.17 ± 0.10  0.090^c^-0.44 | 0.14 ± 0.080  0.12^c^-0.64 | 0.21 ± 0.00  0.21^c^-0.21 | 2.18 |
| Blue mussels, steamed | 0.66 ± 0.25  0.40-1.14 | 0.028 ± 0.018  0.012^c^-0.045 | 1.97 ± 1.4  0.64^c^-4.13 | 0.10 ± 0.041  0.045^c^-0.16 | 1.22 ± 0.47  0.48-1.80 | 0.27 ± 0.073  0.16-0.42 | 0.27 ± 0.12  0.10^c^-0.45 | 0.095 ± 0.15  0.015^c^-0.45 | 0.066 ± 0.042  0.027^c^-0.11 | 4.68 |
| Blue mussels, in brine | 0.76 ± 0.074  0.67-0.84 | 0.037 ± 0.017  0.012^c^-0.045 | 3.72 ± 0.56  3.00-4.17 | 0.25 ± 0.026  0.21-0.27 | 5.22 ± 2.4  2.39-8.14 | 1.14 ± 0.27  0.82-1.46 | 0.87 ± 0.12  0.71-1.01 | 0.070 ± 0.020  0.060^c^-0.10 | 0.12 ± 0.023  0.11^c^-0.15 | 12.2 |
| Blue mussels, pre-packaged | 0.87 ± 0.33 0.63-1.33 | 0.037 ± 0.017 0.012^c^-0.0045 | 4.47 ± 1.3 2.67-5.43 | 0.62 ± 0.28  0.36-0.98 | 8.85 ± 4.3  3.35-13.7 | 2.45 ± 1.2  0.70-3.54 | 1.27 ± 1.0  0.045^c^-2.46 | 0.093 ± 0.065  0.060^c^-0.19 | 0.19 ± 0.17  0.11^c^-0.45 | 18.9 |
| **SCALLOPS** |  |  |  |  |  |  |  |  |  |  |
| Deep sea scallop, raw | 0.25 ± 0.16  0.071-0.56 | 0.012 ± 0.00  0.012^c^-0.012 | 0.10 ± 0.047  0.035-0.17 | 0.058 ± 0.036  0.012^c^-0.099 | 0.019 ± 0.0010  0.016^c^-0.019 | 0.0075 ± 0.00  0.0075^c^-0.0075 | 0.012 ± 0.00  0.012^c^-0.012 | 0.015 ± 0.00  0.015^c^-0.015 | 0.027 ± 0.00  0.027^c^-0.027 | 0.50 |
| Great scallop, raw | 0.023 ± 0.0 0.023^c^-0.023 | 0.023 ± 0.00  0.023^c^-0.023 | 0.053 ± 0.020  0.015^c^-0.088 | 0.023 ± 0.00  0.023^c^-0.023 | 0.038 ± 0.00  0.038^c^-0.038 | 0.018 ± 0.010  0.015^c^-0.048 | 0.046 ± 0.040  0.023^c^-0.13 | 0.030 ± 0.00  0.030^c^-0.030 | 0.053 ± 0.00  0.053^c^-0.053 | 0.31 |
| **SHRIMPS** |  |  |  |  |  |  |  |  |  |  |
| Northern shrimps, peeled | 0.054 ± 0.036  0.012^c^-0.16 | 0.012 ± 0.00  0.012^c^-0.012 | 0.075 ± 0.051  0.0080^c^-0.20 | 0.017 ± 0.013  0.012^c^-0.060 | 0.069 ± 0.053  0.019^c^-0.21 | 0.043 ± 0.028  0.0080^c^-0.11 | 0.022 ± 0.013  0.012^c^-0.064 | 0.016 ± 0.0040  0.015^c^-0.030 | 0.029 ± 0.0070  0.027^c^-0.053 | 0.34 |
| Northern shrimps, unpeeled | 0.39 ± 0.16  0.14-0.57 | 0.012 ± 0.00  0.012^c^-0.012 | 0.20 ± 0.075  0.086-0.31 | 0.17 ± 0.089  0.012^c^-0.25 | 1.13 ± 0.48  0.63-1.89 | 0.67 ± 0.24  0.35-0.99 | 0.39 ± 0.16  0.26-0.72 | 0.089 ± 0.080  0.015^c^-0.24 | 0.043 ± 0.032  0.027^c^-0.11 | 3.12 |
| Northern shrimps, in brine | 0.035 ± 0.0090  0.012^c^-0.054 | 0.012 ± 0.00  0.012^c^-0.012 | 0.047 ± 0.030  0.0040^c^-0.13 | 0.012 ± 0.00  0.012^c^-0.012 | 0.019 ± 0.00  0.019^c^-0.019 | 0.016 ± 0.018  0.0075^c^-0.093 | 0.012 ± 0.0040  0.012^c^-0.034 | 0.015 ± 0.00  0.015^c^-0.015 | 0.027 ± 0.00  0.027^c^-0.027 | 0.20 |
| **CRABS** |  |  |  |  |  |  |  |  |  |  |
| Brown crab, claw meat | 0.060 ± 0.035  0.012^c^-0.14 | 0.012 ± 0.00  0.012^c^-0.012 | 0.011 ± 0.014  0.0075^c^-0.067 | 0.012 ± 0.00  0.012^c^-0.012 | 0.042 ± 0.039  0.019^c^-0.15 | 0.022 ± 0.021  0.0075^c^-0.082 | 0.017 ± 0.015  0.012^c^-0.065 | 0.015 ± 0.00  0.015^c^-0.015 | 0.027 ± 0.00  0.027^c^-0.027 | 0.22 |
| Brown crab, brown meat | 10.5 ± 3.8  3.29-17.0 | 0.038 ± 0.014  0.012^c^-0.045 | 0.50 ± 0.28  0.18-1.16 | 1.60 ± 1.0  0.47-3.34 | 16.3 ± 13  2.89-38.1 | 7.93 ± 6.3  1.60-20.9 | 4.08 ± 3.1  0.77-10.6 | 1.08 ± 0.83  0.20-2.46 | 0.72 ± 0.56  0.11^c^-1.79 | 42.8 |
| Stuffed brown crab shells | 4.10 ± 2.7  0.22-8.25 | 0.026 ± 0.018  0.012^c^-0.045 | 0.14 ± 0.085  0.030^c^-0.30 | 0.51 ± 0.24  0.20-0.86 | 9.06 ± 8.0  3.55-26.2 | 2.65 ± 0.84  1.56-3.87 | 1.69 ± 0.34  1.18-2.25 | 0.36 ± 0.15  0.060^c^-0.50 | 0.22 ± 0.077  0.11^c^-0.33 | 18.8 |
| Snow crab, leg meat | 0.026 ± 0.014  0.012^c^-0.040 | 0.012 ± 0.00  0.012^c^-0.012 | 0.015 ± 0.0080  0.0080^c^-0.024 | 0.012 ± 0.00  0.012^c^-0.012 | 0.019 ± 0.00  0.019^c^-0.019 | 0.025 ± 0.0080  0.016-0.034 | 0.012 ± 0.00  0.012^c^-0.012 | 0.015 ± 0.00  0.015^c^-0.015 | 0.027 ± 0.00  0.027^c^-0.027 | 0.16 |
| Snow crab, hepatopancreas | 5.35 ± 2.1  2.90-7.91 | 0.012 ± 0.00  0.012^c^-0.012 | 4.28 ± 1.2  2.66-5.66 | 2.81 ± 1.2  1.61-4.14 | 60.9 ± 27  23.4-95.6 | 47.1 ± 20  19.6-70.8 | 27.4 ± 14  12.5-42.5 | 12.7 ± 7.7  4.52-21.9 | 7.76 ± 5.7  2.03-16.5 | 168.3 |
| **LOBSTERS & CRAYFISH** |  |  |  |  |  |  |  |  |  |  |
| Crayfish, tails, boiled | 0.79 ± 0.11  0.71-0.86 | 0.012 ± 0.0  0.012^c^-0.012 | 0.0080 ± 0.00  0.0080^c^-0.0080 | 0.066 ± 0.078  0.012^c^-0.12 | 0.11 ± 0.13  0.019^c^-0.20 | 0.089 ± 0.087  0.028-0.15 | 0.057 ± 0.045  0.025-0.089 | 0.015 ± 0.00  0.015^c^-0.015 | 0.027 ± 0.00  0.027^c^-0.027 | 1.16 |
| Norway lobster, white meat, raw | 0.021 ± 0.016  0.012^c^-0.039 | 0.012 ± 0.0060  0.012^c^-0.012 | 0.36 ± 0.32  0.0075^c^-0.61 | 0.012 ± 0.00  0.012^c^-0.012 | 0.019 ± 0.00  0.019^c^-0.019 | 0.027 ± 0.035  0.0075^c^-0.067 | 0.012 ± 0.00  0.012^c^-0.012 | 0.015 ± 0.00  0.015^c^-0.015 | 0.027 ± 0.00  0.027^c^-0.027 | 0.49 |
| Norway lobster, white meat, boiled | 0.021 ± 0.0070  0.012^c^-0.028 | 0.012 ± 0.0060  0.012^c^-0.023 | 0.0095 ± 0.0040  0.0075^c^-0.015 | 0.014 ± 0.0060  0.012^c^-0.023 | 0.024 ± 0.0090  0.019^c^-0.038 | 0.017 ± 0.0020  0.015^c^-0.019 | 0.018 ± 0.0080  0.012^c^-0.027 | 0.019 ± 0.0070  0.015^c^-0.030 | 0.033 ± 0.013  0.027^c^-0.053 | 0.16 |
| Norway lobster, hepatopancreas, raw | 0.70 ± 0.64  0.30-1.83 | 0.023 ± 0.00  0.023^c^-0.023 | 1.27 ± 0.57  0.015^c^-1.27 | 1.15 ± 0.12  1.06-1.29 | 2.75 ± 2.3  1.16-6.78 | 2.04 ± 2.2  0.81-5.87 | 0.92 ± 0.59  0.45-1.89 | 0.28 ± 0.16  0.20-0.57 | 0.053 ± 0.00  0.053^c^-0.053 | 9.18 |
| American lobster, white meat, boiled | 0.12 ± 0.14  0.012^c^-0.35 | 0.013 ± 0.0040  0.012^c^-0.023 | 0.038 ± 0.027  0.0075^c^-0.073 | 0.079 ± 0.098  0.012^c^-0.27 | 0.091 ± 0.090  0.019^c^-0.26 | 0.044 ± 0.036  0.0075^c^-0.11 | 0.034 ± 0.035  0.012^c^-0.10 | 0.023 ± 0.018  0.015^c^-0.060 | 0.040 ± 0.032  0.027^c^-0.11 | 0.48 |
| American lobster, hepatopancreas, boiled | 2.16 ± 3.3  0.22-6.02 | 0.026 ± 0.017  0.012^c^-0.045 | 0.23 ± 0.16  0.11-0.41 | 2.06 ± 1.3  1.19-3.58 | 1.24 ± 0.25  0.96-1.44 | 0.51 ± 0.093  0.43-0.61 | 0.45 ± 0.11  0.35-0.57 | 0.035 ± 0.023  0.015^c^-0.060 | 0.079 ± 0.045  0.027^c^-0.11 | 6.79 |
| European lobster, white meat, raw | 0.055 ± 0.022  0.037-0.13 | 0.012 ± 0.00  0.012^c^-0.012 | 0.044 ± 0.034  0.016^c^-0.15 | 0.012 ± 0.0  0.012^c^-0.012 | 0.063 ± 0.052  0.019^c^-0.22 | 0.32 ± 0.46  0.012^c^-1.58 | 7.97 ± 1.2  0.012^c^-56.0 | 0.11 ± 0.015  0.015^c^-0.79 | 0.027 ± 0.00  0.027^c^-0.027 | 8.61 |
| European lobster, hepatopancreas, raw | 0.52 ± 0.29  0.18-1.18 | 0.012 ± 0.00  0.012^c^-0.012 | 0.021 ± 0.010  0.0075^c^-0.035 | 0.12 ± 0.036  0.049-0.15 | 1.15 ± 0.88  0.40-3.21 | 0.84 ± 0.69  0.22-2.04 | 2.62 ± 2.4  0.34-6.60 | 0.097 ± 0.050  0.031-0.17 | 0.027 ± 0.00  0.027^c^-0.027 | 5.40 |
| European lobster, white meat, boiled | 0.054 ± 0.035  0.012^c^-0.16 | 0.012 ± 0.00  0.012^c^-0.012 | 0.035 ± 0.026  0.0075^c^-0.096 | 0.012 ± 0.0  0.012^c^-0.012 | 0.051 ± 0.048  0.019^c^-0.20 | 0.048 ± 0.033  0.020-0.12 | 0.032 ± 0.035  0.012^c^-0.14 | 0.015 ± 0.0  0.015^c^-0.015 | 0.027 ± 0.00  0.027^c^-0.027 | 0.28 |
| European lobster, hepatopancreas, boiled | 0.30 ± 0.19  0.029-0.55 | 0.012 ± 0.00  0.012^c^-0.012 | 0.021 ± 0.010  0.0075^c^-0.037 | 0.091 ± 0.097  0.012^c^-0.29 | 0.55 ± 0.35  0.046-1.08 | 0.33 ± 0.26  0.039-0.92 | 0.23 ± 0.11  0.012^c^-0.35 | 0.038 ± 0.034  0.015^c^-0.10 | 0.027 ± 0.00  0.027^c^-0.027 | 1.67 |

^a^ For individual values below the limit of quantification (LOQ), the LOQ value was divided by two (medium-bound LOQ: LOQ/2) to enable the calculation of the mean and standard deviation (SD).
^b^ All values for all shellfish products were below the limit of quantification (LOQ) for ß, ϒ-2H-K_1_. The SD varied due to the differences in the LOQ caused by variations in analytical conditions (see Table 2, Appendix B).
^c^ At least the minimum value presented corresponds to the LOQ divided by two, as the minimum value was <LOQ. For some vitamers, such as dihydro K_1_, the maximum value presented may also correspond to the LOQ divided by two, as all values were <LOQ.  **Abbreviations**: ß, ϒ-2H-K_1_**:** beta, gamma-dihydrophylloquinone; LOQ: limit of quantification; MK: menaquinone; SD: standard deviations.
